# Candidate genes at the *Rmi1* locus for resistance to *Meloidogyne incognita* in soybean

**DOI:** 10.1007/s00122-025-05065-w

**Published:** 2025-10-29

**Authors:** Kelly Goode, Tatyana Nienow, Wayne Parrott, Zenglu Li, Melissa G. Mitchum

**Affiliations:** 1 Institute of Plant Breeding, Genetics, and Genomics, University of Georgia, Athens, GA USA; 2 Department of Crop and Soil Sciences, University of Georgia, Athens, GA USA; 3 Department of Plant Pathology, University of Georgia, Athens, GA USA

## Abstract

**Key message:**

The RKN resistance locus *Rmi1* was fine-mapped to two genes on chromosome 10, a glycosyl hydrolase family 9 β-1,4-endoglucanase gene and a type I pectin methylesterase gene.

**Abstract:**

Root-knot nematodes (*Meloidogyne* spp.) are a serious threat to soybean production in the southeast USA, with yield losses of more than $165 million in 2023. Development and deployment of resistant soybean cultivars is the most effective strategy for managing these nematode pests; however, the identity of the resistance genes and underlying mechanism of resistance remains obscure. An additive resistance gene, *Resistance to M. incognita-1* (*Rmi1*), to the predominant species, was first identified in soybean cultivar Forrest but never mapped to a genomic region. Multiple mapping studies have identified a major quantitative trait locus (QTL) with additive action on chromosome 10. In this study, a population consisting of 170 F_2:3_ families derived from a cross of Bossier (susceptible) × Forrest (resistant) was initially used to confirm that *Rmi1* is in the chromosome 10 QTL. Subsequently, 884 F_5:6_ recombinant inbred lines (RILs) derived from the same cross were used to fine-map the *Rmi1* causal gene(s) to two genes – a β-1,4-endoglucanase (*Glyma.10G017000*, *EG*) and a pectin methylesterase/methylesterase inhibitor (*Glyma.10G017100*, *PME1*). Both gene candidates have the potential to play a role in the resistance response to *M. incognita.* Both gene promoters harbor SNPs and indels and the encoded proteins exhibit amino acid polymorphisms, including a premature stop in *PME1* of resistant soybeans. Additionally, both genes show a higher expression level in susceptible roots compared to resistant roots in the absence of infection. This suggests that *Rmi1* may confer one or more pre-existing differences related to cell wall modification in soybean roots, ultimately leading to a decrease in susceptibility.

**Supplementary Information:**

The online version contains supplementary material available at 10.1007/s00122-025-05065-w.

## Introduction

Root-knot nematodes (RKN) are the most economically damaging pathogen threat to soybean production in the southeast US, accounting for more than an estimated $165 million yield loss (2.4%) in 2023 (Crop Protection Network 2024). A 2021 soil survey of Georgia soybean fields identified RKN in 49% of tested samples (Mitchum et al. 2023). *Meloidogyne incognita* was by far the most prevalent RKN species identified, being found in 80% of RKN positive samples. RKN infective second-stage juveniles (J2) hatch from eggs in the soil and are attracted to host roots. J2s enter the root at the elongation zone using physical force by the stylet and secretion of cell wall degrading enzymes (Holbein et al. 2016; Huang and Maggenti 1969; Jaubert et al. 2002; Rosso et al. 1999), then travel intercellularly to the root tip through the cortex, bypassing the endodermis and then migrating back up the vascular cylinder (Abad et al. 2009; Jones and Goto 2011). Once the J2 chooses an initial feeding cell, usually a pro-vascular cell, the nematode becomes sedentary and moves its stylet between five to nine nearby cells to feed (Taylor and Sasser 1978). The nematode secretes effectors into these cells to manipulate cellular function, leading to the formation of giant cells, which expand up to 400 times the size of a normal root cell and undergo repeated rounds of mitosis without cell division to become multinucleate (Caillaud et al. 2008, Jones et al. 2011). Giant cell formation requires substantial manipulation of the plant cell wall. The cell wall must be made more flexible to allow expansion while concurrently accelerating production of cell wall components. In addition to giant cells becoming multinucleate, the abundance of other organelles such as mitochondria, vacuoles, Golgi bodies, and endoplasmic reticulum increases (Jones 1981; Jones and Payne 1978). Giant cells can have a 600 × increase in gene copy number from the increased number of nuclei, many of which also increase in ploidy (Huang et al. 1969; Wiggers et al. 1990). The cells neighboring the giant cells are also stimulated to divide to give rise to the gall or “knot”, from which RKN get their name. Giant cells share characteristics with functional transfer cells (Moens et al. 2009) and serve as the sole nutrition source for the nematode for the rest of its life. RKN infection in the roots can cause severe phenotypic damage aboveground. Plants can wilt or show symptoms of nutrient deficiencies due to the decrease in water and nutrient transport from the roots. The primary methods for managing RKN are crop rotation, nematicides, and genetic resistance. The wide host range of RKN, especially that of *M. incognita*, lessens the effectiveness of crop rotation. Nematicides are available and effective but are less desirable due to their high toxicity. Genetic resistance is available, effective, economical, and environmentally friendly, making it the best option for RKN management.

The first locus for resistance to RKN in soybean was identified from a cross between the susceptible cv. Bossier and the resistant cv. Forrest (Luzzi et al. 1994). Forrest was selected for the study because, at that time, it represented the highest level of RKN resistance commercially available. Screening of F_2_ plants showed a 1:2:1 segregation ratio of resistant:intermediate:susceptible responses to *M. incognita*, indicating a single additive gene conferred RKN resistance in Forrest, named *Resistance to M. incognita-1 (Rmi1)*. Pedigree analysis of 48 soybean lines showed that Forrest and its relatives initially inherited resistance from cv. Palmetto (Ha et al. 2004). Additional screening later identified an accession, PI 96354, which shows a higher level of resistance compared to Forrest. PI 96354 has fewer galls and eggs per root system than Forrest after *M. incognita* infection (Luzzi et al. 1987). The value of PI 96354 was further confirmed in a field microplot study, showing that PI 96354 has < 1% yield suppression under nematode pressure, with cvs. Bossier and Forrest showing 97 and 12% yield suppression, respectively (Herman et al. 1990). *Rmi1* confers resistance to *M. incognita* by triggering the emigration of J2s from roots in the days following penetration (Herman et al. 1991). The highly resistant PI 96354 has additional resistance stemming from delayed development of the nematodes remaining in the root and from decreased fecundity of the few female nematodes making it to egg production (Herman et al. 1991).

**Fig. 1:**
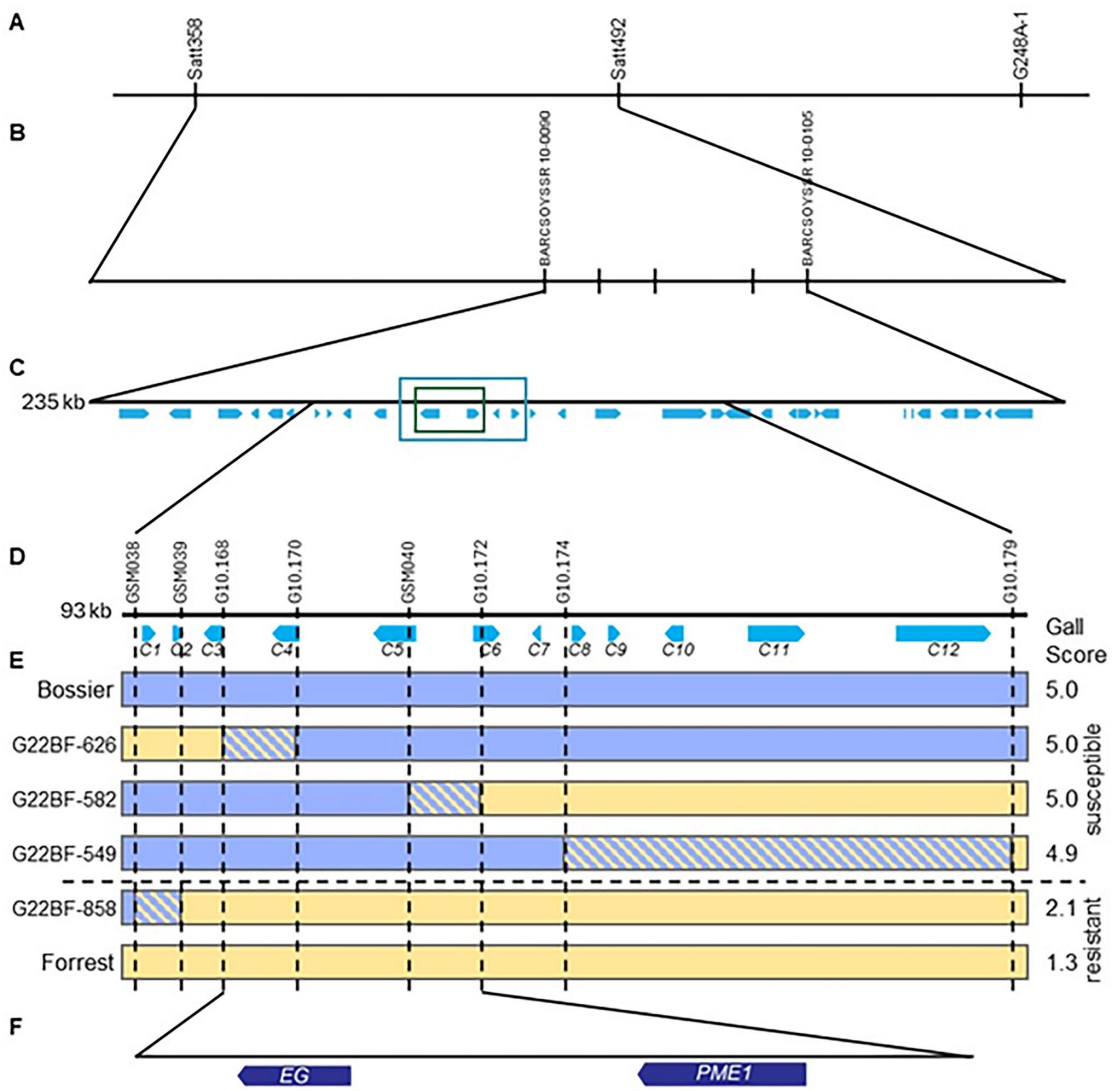
A diagram illustrating the fine-mapping process leading to the identification of two candidate genes at the *Rmi1 locus* for resistance to *M. incognita.* **A** Chromosome 10 QTL region identified by Tamulonis et al. 1997 and Li et al. 2001; **B** Markers used by Pham et al. 2013 to fine-map the chromosome 10 QTL in PI 96354; **C** 235-kb genomic region identified by Pham et al. 2013. The Wm82.a6.v1 reference genome (displayed) annotates 32 genes in this region. The blue box represents the 30-kb region identified by Xu et al. 2013, while the green box highlights the genes identified by Passianotto et al. 2017; **D** Markers used for fine-mapping the candidate region in this study; **E** KASP genotyping results of recombinant F_5:6_ lines along with their corresponding phenotype data. Root systems were scored on a scale of 1–5, with 1 = resistant and 5 = susceptible. The gall scores presented are the mean values across all replicates. **F** The 27.5-kb fine-mapped genomic region between markers G10.168 and G10.172 includes two genes – *Glyma.10G017000* (*EG*) and *Glyma.10G017100* (*PME1*)

Restriction fragment length polymorphism mapping of *M. incognita* resistance using 101 F_2:3_ lines derived from a cv. Bossier x PI 96354 cross identified a major additive QTL on chromosome (Chr) 10 and a minor dominant QTL on Chr 18 (Tamulonis et al. 1997). Since the Chr 10 QTL was predicted to have additive action, it was hypothesized that this region corresponded to *Rmi1* from Forrest but was never independently verified. This study was hampered by a lack of polymorphic markers in the mapped region. Li et al. (2001) utilized the same mapping population to more finely map the region once SSR markers were available. This study identified a major QTL between markers Satt358 and Satt492 (Fig. 1A). The higher marker density increased the percentage of variation explained from 31 to 56%. After the sequencing of the soybean genome revealed more SSR markers (Schmutz et al. 2010), a population of 188 F_5:6_ recombinant inbred lines (RILs) derived from a cv. Bossier × PI 96354 cross were evaluated for *M. incognita* resistance and genotyped using 28 SSR markers between Satt358 and Satt492. This process identified a 235-kb region bordered by BARCSOYSSR10-0090 and BARCSOYSSR10-0105 (Fig. 1B and C), an interval containing 30 gene models (Pham et al. 2013). Xu et al. (2013) sequenced 246 RILs derived from a cv. Magellan × PI 438489B cross to create a bin map, and a major QTL was identified inside the Pham et al. (2013) candidate region (Fig. 1C, blue box). Passianotto et al. (2017) used 188 PIs and two resistant and three susceptible cultivars as a panel in a genome-wide association study (GWAS) and identified five SNPs significantly associated with resistance within the region overlapped by that of Pham et al. (2013) and Xu et al. (2013) (Fig. 1C, green box).

Since Forrest and PI 96354 share the J2 emigration aspect of resistance and the major QTL on Chr 10 also shows additive inheritance (Herman et al. 1991), *Rmi1* is likely located in the region of the Chr 10 QTL. This study used a Forrest-derived RIL population to confirm that *Rmi1* is in the chromosome 10 QTL and to narrow the genomic region previously identified by Pham et al. (2013) in PI 96354.

## Materials and methods

A. 2.1. Plant materials and population development.

A cross was made between soybean cultivars Bossier and Forrest in the summer of 2020 at the University of Georgia (UGA) J. Phil Campbell Research and Education Center in Watkinsville, GA. Forrest, a maturity group (MG) V cultivar (Hartwig and Epps 1973), exhibits resistance to *M. incognita* while Bossier, an MG VIII cultivar, is highly susceptible to this nematode (Luzzi et al. 1994). The F_1_ plants were grown at the USDA winter nursery in Isabela, Puerto Rico, during the 2020–2021 winter. Subsequently, F_2_ plants were grown at the UGA Iron Horse Farm in 2021. At this time, 170 F_2_ plants were pulled and threshed individually to form an F_2:3_ population. In addition, in the fall of 2021, pods were taken from each F_2_ plant for advancement through single seed descent (Poehlman and Sleper 1995) for two generations at the Illinois Crop Improvement Association (ICIA) nursery in San Diaz, Puerto Rico, during the 2021–2022 season. The resulting F_5_ seeds were planted at the UGA Iron Horse Farm in the summer of 2022. In the fall, 884 F_5_ plants were harvested and threshed individually to form a F_5:6_ RIL population that was used for genotyping and phenotyping.

Additionally, two RILs, ExF63 and ExF67, derived from a cross of Forrest (resistant) × Essex (susceptible) (Lightfoot et al. 2005), were also used to evaluate gene expression. Both RILs were derived from the F_5_ generation. ExF63 is resistant, while ExF67 is susceptible to *M. incognita*. Based on 50 k SNP analysis, the RILs exhibit 84% similarity in their genomes (Gamage and Mitchum, pers. comm.).

B. 2.2. Evaluation of *M. incognita* resistance.

A *M. incognita* race 3 population was used in this study (Hussey and Boerma 1981). This population was maintained at UGA on eggplant cv. Black Beauty with yearly passage on susceptible soybean cv. Williams 82 to preserve virulence. Eggs were extracted for inoculation according to Hussey and Barker (1973). For inoculation with J2s, eggs were surface-sterilized in 0.02% sodium azide for 20 min, washed thoroughly with water, then placed on a hatching sieve in water supplemented with antibiotics (1.5 mg mL^−1^ gentamycin and 0.05 mg mL^−1^ nystatin) at 27 °C for three days.

Seeds were germinated in CYG germination pouches (Mega International, Roseville, MN) for three days at 25 °C in the dark. After three days, three seedlings of each of the 170 F_2:3_ families, along with the parents, were planted into Cone-Tainers™ containing heat-sterilized loamy sand soil. Plants were grown in a greenhouse in Athens, GA kept at 75–87 °C with a 16-h day/8-h night light cycle with supplemental lighting from 400-W high pressure sodium lamps. Each plant was inoculated with 2,500 M*. incognita* eggs 11 days after planting. Six weeks after inoculation, the root systems were rinsed free of soil, and gall severity was scored from 1 to 5, with 1 being the galling level of the resistant parent Forrest and 5 being the galling level of the susceptible parent Bossier. The susceptible cv. Williams 82 was also included as a susceptible check.

An additional screen of 21 F_2:3_ families was conducted due to their segregation at the GSM039 locus, the marker currently used for marker-assisted selection at UGA (Pham et al. 2013). This screen was conducted as described above, modified to screen 12 replicates for each family.

Greenhouse screening of selected F_5:6_ Bossier × Forrest RILs was conducted using the procedure described above with the following modifications – the plants were inoculated with 3,000 M*. incognita* eggs three days after planting, and roots were phenotyped eight weeks after inoculation. Sequentially, four independent assays were conducted, with five to 12 replicates planted, depending on the assay. Each screen contained equal replicates of both parents for use as checks to score root systems. In screens of selected F_5:6_ RILs, the galling of each plant was scored by two individuals, and the mean of the two scores was used as the phenotype for the plant.

C. 2.3. RIL genotyping

For initial genotypic screening of 884 F_5:6_ Bossier × Forrest RILs, DNA was extracted from five pooled seeds of each line using a modified protocol described by (Edwards et al. 1991). Briefly, five seeds were incubated in 2 mL Edwards extraction buffer (200 nM Tris pH 7.5, 250 nM NaCl, 25 mM EDTA, 0.5% SDS) at 65 °C for 4 h, then overnight at 4 °C. After incubation, NaCl was added to a final concentration of 250 mM. DNA was precipitated with isopropanol, washed with ethanol, and suspended in 100 µl of dH_2_O.

The Kompetitive allele-specific PCR (KASP) marker, GSM039, was used to genotype all samples (Pham et al. 2013). Additional KASP markers were designed for high-density coverage near the overlapping region identified in prior mapping studies, using Geneious Prime 2022 and the Wm82.a6.v1 reference genome (Espina et al. 2024) (Table 1, Supplemental Table 1). Sanger sequencing reads of candidate genes in Forrest and Bossier were used in selection of SNPs for KASP targeting. For regions where Sanger reads were not available, publicly available Illumina data were used (Supplemental Table 2, Supplemental File 1). Illumina reads were retrieved using the SRA toolkit v3.0.1 (Sayers et al. 2022), aligned to the Wm82.a6.v1 reference genome using Bowtie2 v.2.5.2 (Langmead and Salzberg 2012), and the candidate region sequences were extracted using SAMtools v1.16.1 (Danecek et al. 2021).

**Table 1:** Candidate genes present in the region included in the new KASP marker design on chromosome 10 based on Wm82.a6.v1 reference genome

ID	Gene	NCBI ID	Phytozome Annotation	NCBI RefSeq Annotation
C1	*Glyma.10G016600*	*1.01E* + *08*	pollen ole e 1 allergen and extensin family protein	uncharacterized LOC100527907
C2	*Glyma.10G016700*	*1.01E* + *08*	pollen ole e 1 allergen and extensin family protein	protein seed and root hair protective protein
C3	*Glyma.10G016832*	*1.07E* + *08*	no functional annotation available	uncharacterized LOC106794947, ncRNA
C4	*Glyma.10G017000*	*1.01E* + *08*	endo-1,4-β-glucanase	endoglucanase 17
C5	*Glyma.10G017100*	*1.01E* + *08*	pectin methylesterase/pectin methylesterase inhibitor	pectinesterase-like
C6	*Glyma.10G017200*	*1.01E* + *08*	pectin methylesterase/pectin methylesterase inhibitor	probable pectinesterase/pectinesterase inhibitor 41
C7	*Glyma.10G017300*	*1.01E* + *08*	plant invertase/pectin methylesterase inhibitor	pectinesterase inhibitor 3
C8	*Glyma.10G017400*	*1.01E* + *08*	unknown function; At5g01610-like	uncharacterized LOC100500182
C9	*Glyma.10G017500*	*1.01E* + *08*	unknown function; At5g01610-like	uncharacterized protein At5g01610
C10	*Glyma.10G017600*	*1E* + *08*	late embryogenesis abundant protein, LEA-5	uncharacterized LOC100306369
C11	*Glyma.10G017700*	*1.01E* + *08*	poly(ADP-ribose) polymerase	poly [ADP-ribose] polymerase 2-A
C12	*Glyma.10G017800*	*1.01E* + *08*	gamma-tubulin complex component protein	gamma-tubulin complex component 4 homolog

KASP was performed using low ROX KASP-TF Master Mix (LGC Biosearch Technologies) or PACE Genotyping Master Mix (3cr Bioscience) in 4-µL reactions, with ~ 20 ng genomic DNA using a Lightcycler 480 II instrument (Roche) or Bio-Rad thermal cycler (Hercules, CA, USA). Fluorescence of the plates was read with a Tecan Infinite M1000 Pro (Tecan Trading AG, Switzerland). Allele calls were made using KlusterCaller (LGC Biosearch Technologies).

For genotyping plants, a square centimeter section of a young leaf was taken from each plant two weeks after planting. The samples were collected in 1.4-mL tubes placed in a 96-well latch rack, dried, then ground using a Genogrinder 2010 (SPEX SamplePrep, Metuchen, NJ) with a single bead per tube. DNA was extracted using a CTAB method. Ground tissue was incubated in 800 µL extraction buffer (2% CTAB, 1.4 M NaCl, 100 mM Tris–HCl pH 8, 20 mM EDTA, 0.1% β-mercaptoethanol, 0.34% PVP-40) at 65 °C for 2 h, then centrifuged at 3,600 rpm for 10 min. The supernatant was mixed 1:1 with isopropanol, incubated at − 20 °C for 2 h, then centrifuged at 3,600 rpm for 15 min. The pellet was washed in 70% ethanol and resuspended in 100 µl of dH_2_O.

D. 2.4. Statistical analysis.

Statistical analysis of the F_2:3_ screen was conducted using the base R Pearson’s chi-square function and generalized linear regression of the gall scores and genotyping results of individual plants in R (R Core Team 2024).

For analysis of the F_5:6_ screens, the genotyping results of the 884 F_5:6_ RILs were used to generate a genetic linkage map with KASP markers. The marker distance was calculated using a regression method with Kosambi’s function in JoinMap® 5 (Van Ooijen 2018). The genotyping and phenotyping results of the final greenhouse screen were used for MQM mapping in mapQTL® 7 (Van Ooijen 2023). Automatic cofactor selection was used to identify cofactors for MQM mapping (*p* = 0.05). The LOD threshold was determined using a permutation test with 1,000 permutations (*p* = 0.01). All graphs were made in R v4.4.0 using ggplot2 (Wickham 2016).

E. 2.5. Gene expression analysis.

*EG* and *PME1* expression in root tissue was evaluated in a previously generated RNAseq dataset (Goode and Mitchum 2025) that used soybean RILs ExF63 and ExF67 to profile soybean gene expression changes between nematode-inoculated (with RKN J2 suspended in agarose) and mock-inoculated (with agarose only) roots at 2 and 4 days-post-inoculation (dpi). Sanger sequencing of *EG* and *PME1* in these RILs confirmed that the gene sequences in ExF63 are identical to that of its resistant parent Forrest, while the sequences of ExF67 are identical to that of its susceptible parent Essex. RNAseq reads that mapped to *EG* and *PME1* were extracted using SAMtools v1.17 (Danecek et al. 2021) and visualized using Geneious Prime 2024. EdgeR (Robinson et al. 2010) was used to quantify gene expression using Illumina reads. Results were validated by quantitative RT-PCR (qPCR). Previously, collected root tissue was ground using a liquid nitrogen-chilled porcelain mortar and pestle, then RNA extracted using the QIAGEN Plant RNeasy Mini Kit (QIAGEN, Hilden, Germany). Total RNA (500 ng) was used for cDNA synthesis using PrimeScript 1 st Strand cDNA Synthesis Kit (Takara Bio USA, Mountain View, CA). Sanger sequencing was performed by Genewiz (Azenta Life Sciences, South Plainfield, NJ). Quantitative RT-PCR (qPCR) was performed on a Bio-Rad CFX Real-Time System using PowerUp SYBR Master Mix (Applied Biosystems) according to the manufacturer’s recommendation. The primers used for qPCR are listed in Supplemental Table 3. The reference genes *GmELF1A* and *GmCONS7* were used to normalize expression (Libault et al. 2008; Miranda et al. 2013). Expression was calculated using Bio-Rad CFX Maestro 1.1 (Bio-Rad, Hercules, CA).

To further analyze *EG* expression in root tissues of a comparable developmental stage, root tips were collected from multiple susceptible and resistant genotypes, including Williams 82, Essex, Forrest, ExF67, ExF63, and PI 96354. Briefly, seeds were surface-sterilized by placing in glass Petri dishes inside a glass bell jar desiccator with a beaker containing 100 mL bleach (8.25% NaOCl) and 3.5 mL concentrated HCl for 16 h. Seeds were then rolled in germination paper and placed upright in a glass beaker with a thin layer of dH_2_O on the bottom, covered in plastic wrap with small holes poked through, and incubated in the dark at 25 °C. After three days, seedlings of equivalent lengths were moved to fresh germination paper in Hoagland’s solution. These germination rolls were kept in a 27 °C growth chamber on a 16-h light (100 µmol·m^−2^·s^−1^)/8-h dark cycle. After two days, the bottom 2 cm of the primary root tip was collected from 10 seedlings of each genotype and bulked for RNA isolation, cDNA synthesis, and qPCR as described above.

F. 2.6. Sequence analysis of the candidate genes.

The full-length transcripts of *EG* and *PMEI* were amplified from cDNA generated from roots of RILs ExF63 and ExF67 with ExTaq (Promega) using gene-specific primers (Supplemental Table 3).

Genomic DNA from Bossier, Essex, PI 96354, Forrest, ExF63, and ExF67 was extracted from leaf tissue punches using a CTAB/CHCl_3_/Isopropanol method. Briefly, finely ground leaf tissue was incubated in CTAB extraction buffer (0.1 M Tris–HCl pH 9, 1.4 M NaCl, 20 mM EDTA, 2% CTAB) and 0.3 mg/µl RNase A at 65 °C for 30 min. An equal amount of isoamyl chloroform was added before a 5 min incubation at 25 °C incubation and centrifuging at 17,900 × g for 4 min. The supernatant was mixed 1:1 with isopropanol and incubated at − 20 °C for 20 min, then centrifuged at 17,900 × g for 15 min. The pellet was washed in 70% ethanol and resuspended in 50 µl of dH_2_O. Both candidate genes were amplified from gDNA using Q5 DNA polymerase (New England Biolabs) according to the manufacturer’s recommendation with the primers listed in Supplemental Table 3. Sanger sequencing was performed by Genewiz.

## Results

G. 3.1. Mapping *Rmi1* to chromosome 10.

The previously designed marker GSM039 has 99% accuracy when predicting soybean resistance to *M. incognita* (Pham et al. 2013). This marker allows distinction between parental genotypes Bossier and Forrest. Bossier genotypes as susceptible and Forrest genotypes as resistant at GSM039.

**Fig. 2:**
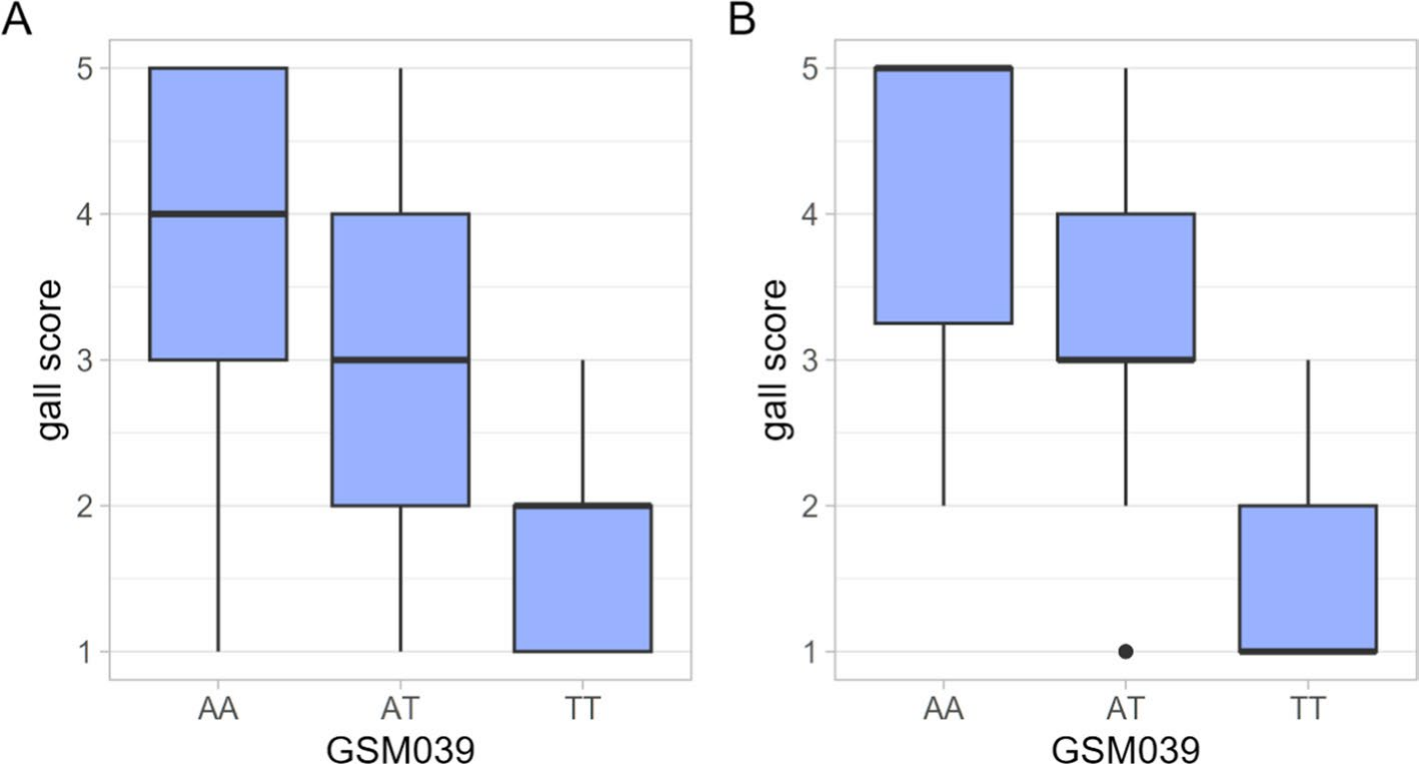
Distribution of gall scores by marker GSM039 genotypes in the F_2:3_ population. **A** gall scores of F_3_ plants in an initial screen of 170 F_2:3_ families; and **B** mean gall score of F_3_ plants in a screen of 21 F_2:3_ families which were found to be segregating at the GSM039 locus. The bars represent the range of scores shown by the plants with the corresponding genotype. The resistant parent, Forrest, is TT, while the susceptible parent, Bossier, is AA at GSM039. Root systems were scored in a scale of 1–5, with 1 being the level of galling on Forrest and 5 being the level of galling on Bossier

Four hundred and sixty-one plants representing 170 F_2:3_ families generated from a cross between Bossier and Forrest were phenotyped (Supplemental Table 4). Eighty-eight plants showed a similar level of resistance to Forrest, 69 plants showed a similar level of susceptibility as Bossier, and the remaining 304 plants had intermediate resistance. Two hundred and five plants were homozygous for the susceptible allele, 165 plants were homozygous for the resistant allele, and 91 plants were heterozygous (Supplemental Table 5). The *Χ*^2^ results indicated that the observed distribution of plants (205:91:165) differed significantly from the expected distribution (*p* = 0.003). Lines genotyped as resistant had a mean gall score of 1.4. Lines genotyped as susceptible had a mean gall score of 4.3. Lines genotyped as heterozygous had a mean gall score of 3.3 (Fig. 2A). A significant (*p* < 0.001) correlation was found between the GSM039 marker and *M. incognita* response, with *R*^*2*^ = 0.5.

A subset of twenty-one F_2:3_ families was selected for additional screening, accounting for those segregating at GSM039 in the initial screen and randomly selected additional F_2:3_ families. A total of 224 F3 plants were phenotyped (Supplemental Table 6). Ninety-six plants genotyped as heterozygous, 66 plants genotyped as susceptible, and 62 plants genotyped as resistant (Supplemental Table 5). Plants genotyped as heterozygous had a mean gall score of 3.3. Plants genotyped as resistant had a mean a gall score of 1.3. Plants genotyped as susceptible had a mean gall score of 4.3 (Fig. 2B). The *Χ*^2^ results indicated that the ratio of observed plants (66:96:62) did not significantly differ from the expected distribution (*p* = 0.094). The correlation between *M. incognita* response and GSM039 was again significant (*p* < 0.001) with *R*^*2*^ = 0.6. Together, these data position *Rmi1* in the chromosome 10 QTL.

H. 3.2. Fine-mapping *Rmi1* using a RIL population.

A 235-kb region associated with *M. incognita* resistance was previously fine-mapped in the resistant PI 96354 (Pham et al. 2013). In that study, three KASP markers, GSM038, GSM039, GSM040, were designed to target three candidate genes of interest in the region, including C1 (*EXT1*, *Glyma.10G016600*), C2 (*EXT2*, *Glyma.10G016700*), and C5 (*PME1, Glyma.10G017100*), respectively, selected based on the presence of SNPs between the parents and differential expression patterns in response to RKN infection (Pham et al. 2013). These markers spanned a 30-kb region containing five candidate genes (Supplemental Table 2; Fig. 1D, C1-C5). In this study, two new markers, G10.168 and G10.170, were designed within the region to target C3 (*unknown*, *Glyma.10G016832*) and C4 (*EG, Glyma.10G017000*), respectively, and allow a marker for each of the five genes (Fig. 1D). Forrest and PI 96354 share an identical sequence with each other in the Chr10 region. Hence, markers were designed for SNPs that could differentiate the Forrest and PI 96354 sequence from sequences present in all three susceptible lines, Williams 82, Essex, and Bossier, thus allowing correlation between the marker and response to *M. incognita*.

To ensure capture of recombinants, three additional markers were designed. Marker G10.172 is located inside *Glyma.10G017200 (PME2)*, while markers G10.174 and G10.179 were designed 9 kb and 65 kb to the right of *Glyma.10G017200*, respectively. These markers are located near candidates C8 (*unknown*, *Glyma.10G017400*) and C12 (*gamma-tubulin*, *Glyma.10G017900*). Thus, of the 235-kb QTL region mapped by (Pham et al. 2013), 90 kb were covered by markers (Fig. 1D). A total of 884 F_5:6_ RILs were genotyped with these 8 markers using DNA extracted from five seeds per line (Supplemental Table 7).

**Fig. 3:**
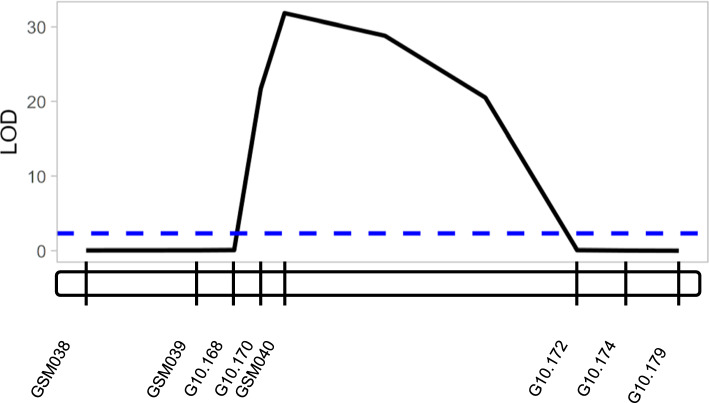
LOD (logarithm of odds) scores along the chromosome 10 candidate gene region for F_5:6_ plants screened for response to *M. incognita*. LOD scores were calculated using the multiple QTL mapping (MQM) method with MapQTL® 7. The blue dashed line indicates a LOD threshold of 2.3, determined using 1000 permutations and ɑ = 0.01

Twelve lines were selected for phenotypic and genotypic confirmation based on identification of single recombinants in all prior greenhouse screens, with eight replicates of each RIL planted (Supplemental Table 8). Four recombination events were identified. Lines G22BF-858, G22BF-626, G22BF-582, G22BF-549 possessed breakpoints between *Glyma.10G016600* and *Glyma.10G06700*, *Glyma.10G016832* and *Glyma.10G017000*, between *Glyma.10G017100* and *Glyma.10G017200*, and between *Glyma.10G017400* and *Glyma.10G017900*, respectively (Fig. 1E). Three RILs phenotyped as susceptible and genotyped as the Bossier alleles at markers G10.170 and GSM040; one RIL(G22BF-858) phenotyped as resistant and genotyped as the Forrest alleles at markers G10.170 and GSM040 A linkage map generated by JoinMap® contained eight markers and spanned 5.9 cM. LOD scores calculated by mapQTL® indicated that the candidate region peaked at marker GSM040 (Fig. 3). This narrowed the *Rmi1* interval to a 27.5-kb region containing only two genes – *Glyma.10G017000* (β-1,4-endoglucanase; *EG*) and *Glyma.10G017100* (pectin methylesterase; *PME1*) (Fig. 1F).

I. 3.3. Candidate gene expression.

*EG* and *PME1* expression in root tissue was evaluated in an RNAseq dataset that used soybean RILs ExF63 and ExF67 to profile soybean gene expression changes between nematode-inoculated (with RKN J2 suspended in agarose) and mock-inoculated (with agarose only) roots at 2 and 4 days-post-inoculation (dpi) (Goode and Mitchum 2025). *EG* read counts were higher in mock-inoculated roots of the susceptible ExF67 (606 reads) compared to mock-inoculated roots of the resistant ExF63 (295 reads) at 2 dpi (Supplemental Fig. 1A and B). A similar pattern of expression was observed for infected roots of ExF67 (610 reads) and ExF63 (238 reads) at 4 dpi (Supplemental Fig. 1C and D). All mapped reads confirmed the predicted splice sites. *EG* had no significant change in expression in response to infection at either timepoint (Supplemental Fig. 3). It did, however, show a significant increase in expression in the mock-inoculated ExF67 root samples when compared to mock-inoculated ExF63 root samples at 2 dpi, indicating a basal expression difference between susceptible and resistant roots (Supplemental Fig. 4A).

**Fig. 4:**
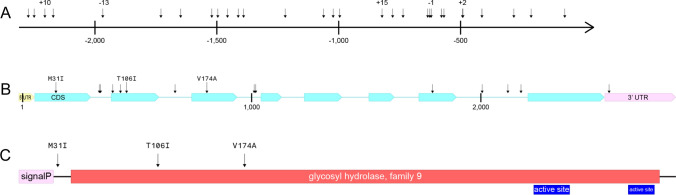
Polymorphisms identified in *Glyma.10G017000* (*EG*) between resistant (Forrest and PI 96354) and susceptible (Williams 82, Essex, and Bossier) lines in **A** the *EG* promoter. Indels are indicated by the size of the insertion or deletions in the resistant allele, **B** the *EG* gene sequence. SNPs resulting in an amino acid change are marked, and **C** the EG protein sequence. Domains are shown as predicted by InterPro (Paysan-Lafosse et al. 2023). The sequences of Forrest and PI 96354 are identical. All polymorphisms are present in both Forrest and PI 96354 when comparing to the susceptible lines Williams 82, Bossier, and Essex. The figures illustrate the difference in the resistant alleles against the Wm82.a6.v1 reference annotation (Espina et al. 2024)

The read counts for *PME1* in the roots of young seedlings were much lower than that of *EG*. Only 13 reads from mock-inoculated roots of the resistant ExF63 (Supplemental Fig. 2A) and six reads from mock-inoculated roots of ExF67 (Supplemental Fig. 2B) mapped to *PME1*. In infected roots, there were only 32 reads from ExF63 (Supplemental Fig. 2C) and 31 reads from ExF67 (Supplemental Fig. 2D) mapped to *PME1* at either time point. The low number of *PME1* reads resulted in the gene being filtered out from the edgeR analysis and therefore could not be quantified. Despite the low number of mapped reads, it was still possible to amplify cDNA of *PME1* from infected root tissue (Supplemental File 2).

To verify the RNAseq results and to achieve greater sensitivity for these low expression genes, qPCR was conducted on cDNA generated from the RNA samples used for the RNAseq analysis. Similar to RNAseq results, neither *EG* nor *PME1* was significantly differentially expressed in response to infection (Supplemental Fig. 3). However, there was a significant difference in *EG* expression between mock-inoculated and infected roots of both genotypes, with ExF67 showing higher expression than ExF63 in both comparisons, underlying the increased expression in the susceptible genotype identified by edgeR (Supplemental Fig. 4A, B, C and D). Expression of *PME1* was higher in susceptible roots when compared to resistant roots, though the only statistically significant difference was between mock-inoculated ExF67 and ExF63 roots at 4 dpi (Supplemental Fig. 4E, F,G and H).

To determine if the lower expression of *EG* in resistant roots compared to susceptible roots was common among resistant genotypes, root tips were collected from susceptible (Williams 82, Essex, ExF67) and resistant (PI 96354, Forrest, and ExF63) lines. The ensuing qPCR analysis showed 3 × increase in expression of *EG* in susceptible lines when compared to resistant lines (Supplemental Fig. 5). Since *EG* shares 95% identity with its close paralog, *Glyma.02G016400* (*GmCel7*), expression of *GmCel7* was also determined. *GmCel7* was not significantly differentially expressed between resistant or susceptible roots (Supplemental Fig. 6).

**Fig. 5:**
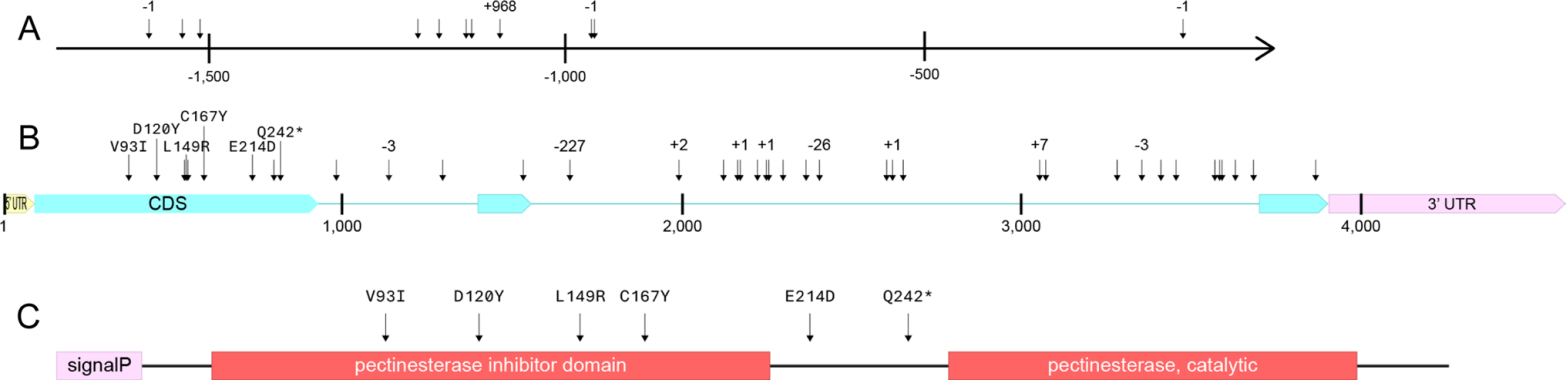
Polymorphisms identified in *Glyma.10G017100* (*PME1*) between the parents Forrest and Bossier. **A** the *PME1* promoter. SNPs are marked with an arrow. Indels are indicated by the size of the insertion or deletions in the resistant allele given above the arrow, **B** the *PME1* gene sequence. SNPs resulting in an amino acid change are marked; and **C** the PME1 protein sequence. Domains are shown as predicted by InterPro (Paysan-Lafosse et al. 2023). All polymorphisms between Forrest and the susceptible Williams 82, Essex, and Bossier are also present in PI 96354. The figures illustrate the difference in the resistant alleles against the Wm82.a6.v1 reference annotation (Espina et al. 2024)

J. 3.4. Candidate gene sequences.

Both *EG* and *PMEI* contain many polymorphisms between resistant and susceptible alleles. To determine the relevance of a polymorphism, the sequences of the resistant Forrest and PI 96354 were compared to those of the susceptible Williams 82, Essex, and Bossier sequences. If a polymorphism was shared between Forrest and PI 96354, but not shared with the susceptible Williams 82, Essex, or Bossier, it was considered to be correlated with resistance.

The *EG* promoter has 23 SNPs, a 10-bp insertion, a 13-bp deletion, a 15-bp insertion, a 1-bp deletion, and a 2-bp insertion in the resistant lines compared to the susceptible Wm82.a6.v1 reference (Fig. 4A). The corresponding alleles from Forrest and PI 96354 also contain three nonsynonymous SNPs (M31I, T106I, V174A), two silent exonic SNPs, five intronic SNPs, a 1-bp intronic deletion, and a 3’ UTR SNP (Fig. 4B and C).

The *PME1* promoters of both Forrest and PI 96354 have a 968-bp insertion approximately 1.1 kb upstream of the 5’ UTR in the resistant lines (Fig. 5A). There are also two single-bp deletions after the 3’ end of this insertion and multiple SNPs before the 5’ end of the insertion. The coding sequence of both Forrest and PI 96354 has five SNPs resulting in several amino acid changes (V93I, D120Y, L149R, C167Y, E214D), one resulting in a premature stop codon (Q242*), and five silent exonic SNPs (Fig. 5B and C). The introns have 227-, 24-, 22-, 3-, and 2-bp deletions, along with three single-bp deletions, 3- and 7-bp insertions, and twenty SNPs between the resistant (Forrest and PI 96354) and susceptible (Williams 82, Essex, and Bossier) lines (Fig. 5B). The predicted splicing patterns of each gene were confirmed by sequencing cDNA (Supplemental File 2).

## Discussion

Genetic resistance is the preferred method of controlling damage from RKN. The first RKN resistance gene, *Mi-1.2*, was mapped in tomato (Milligan et al. 1998). *Mi-1.2* is a canonical *R* gene. Since then, several other *R* genes for RKN have been cloned and even more *R* loci mapped (Goode and Mitchum 2022). For soybean, every published mapping study of *M. incognita* resistance identified a major QTL on chromosome 10, regardless of methods or resistance sources (Fourie et al. 2008; Jiao et al. 2015; Passianotto et al. 2017; Pham et al. 2013; Xu et al. 2013). Unlike other RKN resistance QTL identified in other host plants (Ammiraju et al. 2003; Chen et al. 2007; Harris-Shultz et al. 2019; Wang et al. 2023), this region contains no canonical *R* genes. Rather, there is a variety of genes in this region, including some cell wall-related genes, and others that are not characterized or have unknown functions (Table 1; Pham et al. 2013). This study was able to narrow the *Rmi1 *candidate gene region to two genes in soybean cultivar Forrest.

The use of Forrest for identifying the causal gene(s) in this region was ideal because its resistance is derived from a single locus (Luzzi et al. 1994). This study confirmed that the causal gene(s) for *Rmi1* resistance is in the previously identified QTL on Chr 10 by identifying a significant correlation between the GSM039 marker and the response to *M. incognita* infection in screens of Bossier × Forrest F_2:3_ lines. Confirmation of this location allows linkage of the J2 emigration phenotype demonstrated by Herman et al. (1991) with the Chr 10 QTL. Although the F_2:3_ population used to confirm the *Rmi1* QTL demonstrated segregation distortion among the plants used in the initial screen of 170 F_2:3_ lines, a subset of 21 F_2:3_ lines found to be segregating at GSM039 did not display segregation distortion in a second assay.

The genomic region between *Glyma.10G016600* and *Glyma.10G017300*, the region encompassing genes of interest identified by Pham et al. (2013) and Xu et al. (2013), is highly similar between the resistant genotypes, Forrest and PI 96354. However, they have hundreds of polymorphisms when compared to the same region in the susceptible genotypes, Williams 82 and Bossier. The identical sequence in common between the two resistant genotypes stops abruptly just 5’ to *Glyma.10G017400*. This suggests PI 96354 and Forrest share a common ancestor, resulting in an introgression in this region that ends before *Glyma.10G017400*. The consistent identification of the candidate genes at the 3’ end of this introgression could result from a widespread introgression among resistant soybean lines. Analysis of the lines previously sequenced for GWAS (Passianotto et al. (2017) could offer further insight into this possibility.

Here, the large F_5:6_ RIL population coupled with several new markers spanning the region allowed for capture of multiple recombination events within the Chr 10 QTL. The *Rmi1* interval fine-mapped contains two predicted genes in the Wm82.a6.v1 reference genome – *EG* (*Glyma.10G017000*) and *PME1* (*Glyma.10G017100*)*.* There are two types of PME proteins. PME1 is a Type I PME, containing both a catalytic PME domain and a PME inhibitor domain, while Type II PMEs only contain the catalytic PME domain (Micheli 2001; Pelloux et al. 2007). The PME inhibitor domain is cleaved before a PME is found in the cell wall and likely functions as an intramolecular chaperone of the PME catalytic domain (Micheli 2001). One SNP (GSM040) correlated with resistance results in a premature stop codon before the predicted PME catalytic domain. The NCBI RefSeq database predicts *PME1* (*LOC102665079*) to be a pseudogene (https://www.ncbi.nlm.nih.gov/gene/?term=LOC102665079). Few global soybean RNA transcriptome studies determining expression patterns throughout the plant have identified *PME1* expression and when it has been identified it is at very low levels. Libault et al. (2010) identified *PME1* expression in the soybean shoot apical meristem (SAM). Danzer et al. (2015) identified expression in the soybean seed cotyledon abaxial epidermis. Pelletier et al. (2017) identified expression in the soybean hilum. The maximum level of expression in any of these experiments was 0.33 FPMK or RPMK (Danzer et al. 2015).

Only three other expression studies included in the SoyBase’s gene expression profiling explorer tool (https://legacy.soybase.org/experiments/) identified expression of *PME1* above 1 RPMK or FPMK. Expression was identified at up to 5 FPKM in response to aphid infestation (Brechenmacher et al. 2015), in glabrous and wild-type shoot tips of soybean cv. Clark at up to 2 RPMK (Hunt et al. 2011), and up to 6.6 RPMK in a dehydration and salinity response experiment (Belamkar et al. 2014).

Since *PME1* expression has been identified in some circumstances and a full-length transcript was able to be amplified in this study, it is likely not a pseudogene. Rather, it is a gene that has very low expression and is likely only expressed in specific circumstances and cell types, making it difficult for broader gene prediction programs to identify. The use of scSeq should help clarify its cell-specific expression pattern. *PME1* itself was identified as a candidate in prior mapping studies. It was in the 235-kb region mapped by Pham et al. (2013) and was one of the five genes identified using bin-mapping by Xu et al. (2013), although the authors noted it appeared to be a pseudogene and focused on other genes in the interval. The three most significant SNPs identified by Passianotto et al. (2017) were identified inside *PME1*, while the other two significant SNPs were located in the gene directly downstream of *PME1*, *Glyma.10G017200*.

In plants, PMEs typically demethylesterify (DM) pectin in a linear fashion, acting on chains of homogalacturonan (HG), the most abundant component of pectin (Wolf et al. 2009). This process releases methyl groups from the HG, leaving a binding site for Ca^2+^ ions. The connection of HG chains through Ca^2+^ binding leads to “egg-box” structures that rigidify the cell wall (Micheli 2001; Molina et al. 2024; Wormit and Usadel 2018). If Ca^2+^ binding does not occur, the HG chains remain open to pectin-degrading enzymes, like polygalacturonases (Pelloux et al. 2007). HG degradation by polygalacturonases releases oligogalacturonides that can function as damage-associated molecular patterns (DAMPs) that initiate DAMP-triggered immunity (DPI) (Lionetti et al. 2012; Molina et al. 2024). The level of pectin esterification has been implicated in resistance against wheat fungus (Wiethölter et al. 2003) and liquorice rot in carrots (Le Cam et al. 1994). Arabidopsis cell walls were found to be DM by plant PMEs in response to infection by *Alternaria brassicicola* and *Pseudomonas syringae* (Bethke et al. 2014).

There has also been a direct interaction found between an arabidopsis type I PME and a cellulose binding protein (CBP) secreted by *Heterodera schachtii* (sugar beet cyst nematode) during feeding site formation (Hewezi et al. 2008). The specific binding sites in the interaction were not determined. *M. incognita* infective J2s also secrete a CBP effector (Ding et al. 1998). Mi-CBP could target the catalytic domain in PME1 that is missing from the resistant allele, thus leading to a loss of a susceptibility factor in resistant lines. While establishment of a giant cell requires precise manipulation of the cell wall to rapidly increase the cell size while maintaining wall integrity, J2s travel intercellularly through the middle lamella, a primarily pectin layer, while migrating through the root before selecting an initial feeding cell. RKN secrete other pectin-modifying enzymes while migrating (Huang et al. 2005; Jaubert et al. 2002). Since the exact reason for J2 emigration is unclear, it is not known how close a J2 comes to initiating a feeding site before emigrating. *PME1* could function as a susceptibility factor for either nematode migration or feeding site initiation.

*EG* encodes an EGase belonging to glycosyl hydrolase family 9 (Urbanowicz et al. 2007). EGases hydrolyze bonds in the β-1,4-glucan backbone of cellulose, a compound accounting for 30% of cell wall mass (Ochoa-Villarreal et al. 2012). In other pathogen systems, EGases can act as resistance or susceptibility factors. *SlCel1* and *SlCel2* are needed for tomato susceptibility to *Botrytis cinerea* (Flors et al. 2007). In arabidopsis, plants lacking the EGase KOR1 were more susceptible to *P. syringae* (López-Cruz et al. 2014). Expression of several plant EGases has been identified in giant cells, including the tobacco *NtCel7* and *NtCel8* and the arabidopsis *AtCel1* (Goellner et al. 2001; Sukno et al. 2006). Knockouts of an arabidopsis EGase (*At4g16260*) increased susceptibility to *H. schachtii*, while overexpression of a different arabidopsis EGase (*AtCel6*) in soybean decreased susceptibility to both *Heterodera glycines* (soybean cyst nematode; SCN) and *M. incognita* (Hamamouch et al. 2012; Woo et al. 2014). RNAi knockouts of *GmCel7* decreased susceptibility of soybean to SCN (Woo et al. 2014). Due to the high sequence similarity between *EG* and *GmCel7*, it is likely that EG was also silenced in this study. *EG* expression could, like other GH9 EGases, be induced in giant cells and required for successful giant cell formation, Since the resistance phenotype occurs through emigration of J2s, the nematode needs to sense the lack of the susceptibility factor early enough in the infection process to emigrate. It is unknown how far the J2 can get in initiating a giant cell without becoming sedentary. A low basal level of EG and/or an impaired function of EG in resistant roots could limit the J2’s ability to establish a giant cell thereby triggering the nematode to emigrate to find another root to infect.

*EG* was overlooked in prior studies as a candidate. Pham et al. (2013) set certain criteria when narrowing down the 30 identified genes in the QTL region for sequencing. Genes that were sequenced to identify polymorphisms needed to either have a root-specific expression pattern or were shown to be involved in RKN response previously. Based on the available data at the time, *EG* was not selected for further study. Passianotto et al. (2017) performed a GWAS in which they sequenced nearly 200 lines to identify significant genomic regions and five SNPs in the genes adjacent to *EG* (three in *PME1*, two in *Glyma.10G017200*). All of the SNPs used throughout the genome in their analysis were not reported, just the five that were significant. Thus, it is not clear if SNPs in *EG* were used and not found to be significant or if there were no SNPs in *EG* used in the analysis. Xu et al. (2013) utilized a bin-mapping strategy to fine-map the QTL to a 30-kb region. However, the exact borders of this bin remain unclear, though it is adjacent to *EG*.

While neither of these candidates belongs to a class of canonical *R* genes, there is precedent for nematode resistance in soybean being controlled by non-canonical *R* genes. Notably, neither of the two primary resistance genes against SCN, *Rhg1* and *Rhg4*, are canonical *R* genes. *Rhg1* is controlled by copy number variation of a block of three genes coding for an amino acid transporter, α-soluble N-ethylmaleimide sensitive factor attachment protein (*SNAP18*), and a wound-inducible domain protein (Cook et al. 2012). *Rhg4* encodes a serine hydroxymethyltransferase (Liu et al. 2012). More recently, additional members of the soybean ɑ-SNAP family, including *GmSNAP11* (Shaibu et al. 2022) and *GmSNAP02* (Usovsky et al. 2023) have also been shown to function in SCN resistance.

Narrowing the *Rmi1* locus to two candidate genes significantly decreases the number of genes to be further evaluated for their role in resistance. Both gene candidates have the potential to contribute to the resistance response, since they possess promoter SNPs and indels as well as amino acid polymorphisms in the encoded proteins, including a premature stop in *PME1* of resistant soybeans. Additionally, both genes exhibit a higher level of expression in susceptible roots than in resistant roots. Further functional studies are underway to evaluate the potential role of each gene in resistance to RKN.

Supplemental Files.

Supplemental Table 1. KASP marker primer sequences used for genotyping.

Supplemental Table 2. The NCBI Sequence Read Archive accessions for the Illumina reads corresponding to the soybean lines used in this study for KASP marker design.

Supplemental Table 3. Primers used for mRNA and gDNA amplification for sequencing and qPCR.

Supplemental Table 4. Genotyping and phenotyping results of 170 F_2:3_ families. Genotyping was conducted using the marker GSM039, with TT being the Forrest allele and AA being the Bossier allele. The galling of each root system was scored from 1 (resistant) to 5 (susceptible).

Supplemental Table 5. Summary of genotyping and phenotyping results of the screen of Bossier × Forrest F_2:3_ families. Genotyping was conducted using the marker GSM039, with TT being the Forrest allele and AA being the Bossier allele. The galling of each root system was scored from 1 (resistant) to 5 (susceptible).

Supplemental Table 6. Genotyping and phenotyping results of the screen of 21 F_2:3_ families. Genotyping was conducted using the marker GSM039, with TT being the Forrest allele and AA being the Bossier allele. The galling of each root system was scored from 1 (resistant) to 5 (susceptible).

Supplemental Table 7. Genotyping results of 884 F_5:6_ RILs. Five seeds of each line were bulked together for DNA extraction and genotyping. Cells highlighted in yellow correspond to the alleles donated by Forrest, while the cells highlighted in blue correspond to the alleles donated by Bossier.

Supplemental Table 8. Genotyping and phenotyping results of the greenhouse screen of 12 F_5:6_ Bossier × Forrest RILs. Eight replicates of each line were planted and genotyped with eight KASP markers. Root systems were scored 1 (galling of resistant parent Forrest) to 5 (galling of susceptible parent Bossier).

Supplemental Fig. 1. RNAseq reads aligned to *EG (Glyma.10G017000)* for both replicates and timepoints (2- and 4 dpi). A) ExF63 (resistant) infected roots, B) ExF67 (susceptible) infected roots, C) ExF63 mock-infected roots, and D) ExF67 mock-infected roots.

Supplemental Fig. 2. RNAseq reads aligned to *PME1* (*Glyma.10G017100)* for both replicates and timepoints (2- and 4 dpi). A) ExF63 (resistant) infected roots, B) ExF67 (susceptible) infected roots, C) ExF63 mock-infected roots, and D) ExF67 mock-infected roots.

Supplemental Fig. 3. Gene expression analysis comparing RNAseq samples from mock-inoculated and RKN-infected roots. A-D) *EG*: A) infected to mock-inoculated ExF67 (susceptible) tissue at 2 dpi, B) infected to mock-inoculated ExF67 (resistant) tissue at 4 dpi, C) infected to mock-inoculated ExF63 tissue at 2 dpi and D) infected to mock-inoculated ExF63 tissue at 4 dpi. E–H) *PME1*: E) infected to mock-inoculated ExF67 tissue at 2 dpi, F) infected to mock-inoculated ExF67 tissue at 4 dpi, G) infected to mock-inoculated ExF63 tissue at 2 dpi, and H) infected to mock-inoculated ExF63 tissue at 4 dpi. Since *PME1* was excluded from edgeR analysis due to lack of reads, there are no significance bars for those comparisons.

Supplemental Fig. 4. Gene expression analysis comparing root tissue of ExF67 (susceptible) and ExF63 (resistant). A-D) *EG:* A) ExF67 mock-inoculated to ExF63 mock-inoculated tissue at 2 dpi, B) ExF67 mock-inoculated to ExF63 mock-inoculated tissue at 4 dpi, C) ExF67 RKN-infected to ExF63 RKN-infected tissue at 2 dpi, D) ExF67 RKN-infected to ExF63 RKN-infected tissue at 4 dpi. E–H) *PME1:* E) ExF67 mock-inoculated to ExF63 mock-inoculated tissue at 2 dpi, F) ExF67 mock-inoculated to ExF63 mock-inoculated tissue at 4 dpi, G) ExF67 RKN-infected to ExF63 RKN-infected tissue at 2 dpi, H) ExF67 RKN-infected to ExF63 RKN-infected tissue at 4 dpi. Since *PME1* was excluded from edgeR analysis due to lack of reads, there are no significance bars for those comparisons.

Supplemental Fig. 5. *EG* expression in root tips collected from three susceptible genotypes (Williams 82, Essex, ExF67) and three resistant genotypes (PI 96354, Forrest, ExF63). A 3 × increase in *EG* expression among susceptible lines was observed when compared to the resistant lines.

Supplemental Fig. 6. Gene expression of *GmCel7* (*Glyma.02G016400*) in ExF63 (resistant) and ExF67 (susceptible) root tips.

Supplemental File 1. Code used to retrieve Illumina reads from the NCBI Sequence Read Archive, align reads to the Wm82.a6.v1 reference genome, and extract the sequence of the candidate region.

Supplemental File 2. Alignment of *EG* and *PME1* sequences obtained through Sanger sequencing of the susceptible Essex and Bossier and the resistant Forrest and PI 96354, aligned to the Wm82.a6.v1 reference genome.

## Supplementary Information

Below is the link to the electronic supplementary material.

Supplementary material 1 (TXT 2.1 kb)

Supplementary material 2 (DOCX 34.2 kb)

## Data Availability

The datasets generated during and/or analyzed during the current study are provided as supplemental materials or are available from the corresponding authors on reasonable request.

## Declarations

The authors have filed the following US provisional patent application “*Compositions and methods for increasing resistance to nematodes in plants”*—No. 63/719,027 filed on 11/11/2024.

Correspondence and requests for materials should be addressed to Melissa G. Mitchum and Zenglu Li. Plant material and the nematode population used in this manuscript are available upon request via a material transfer agreement and associated permits.
