# Supplementary material for: Candidate genes at the Rmi1 locus for resistance to Meloidogyne incognita in soybean
Source: Theor Appl Genet. 2025 Oct 29;138(11):286. doi: 10.1007/s00122-025-05065-w (PMC12572094; doi:10.1007/s00122-025-05065-w)
Supplement: Supplementary file 2 — Supplementary material 2 (DOCX 34.2 kb) [file 122_2025_5065_MOESM2_ESM.docx]

# Supplemental File 2 Gene Sequences and Alignments

## *Glyma.10G017000* Promoter Alignment

Since Forrest and PI 96354 sequence are identical, only Forrest is shown in the following alignment. Likewise, as Essex and Bossier sequences are identical, only Essex is shown in the following alignment.

Williams82 GACAAGTAGCAAACTTTTAGGCAATTTTCCAATCAGAGAATATTATTGTCCATTGCAATT
Essex ............................................................
Forrest ...................................T......................A.

Williams82 TGAAAAATAGAAACGGATTCAAGTAAAAACTTACATAATAACAAC----------TAAAA
Essex .............................................----------.....
Forrest .............................................TAAAATAAAA.....

Williams82 TAAAGAAGCCTAAGACAATGACCTTAATTGCTTCGAACTATCCACGGCCTTGGTGCGATG
Essex ............................................................
Forrest ...........................A................................

Williams82 ACACTAGAAATTTTCAAAACGAATTTAAATTGTTAAATTAACTTCAAAATTGAAAATATG
Essex ............................................................
Forrest ............................................................

Williams82 AATTCATTAACTAAAAAACCAAAAAATATCTTTTATTCAATTACGATTTTCATATCAAAA
Essex ............................................................
Forrest ............................................................

Williams82 TTGAACCAAACCATATCATTTATAATATTATTATATTTTTATAAAAGTGACCTTGAAAAG
Essex ............................................................
Forrest ..........................................-------------.....

Williams82 AAATGTCATTTCATTATAATTAAGGATTTAAGGATGTTTATTTTTTAAAAGTTGTTCAAG
Essex ............................................................
Forrest ............................................................

Williams82 GAATATAATATCAAAATCTTACCTTTATACTGAATAAACAAACTCATCCTTATAAACAAC
Essex ............................................................
Forrest ............................................................

Williams82 TACTTTCATATATTTAAGTAATGTAAAAAGTAAACTAATTAATTATCAAAGAAGCTAAAT
Essex ............................................................
Forrest ............................................................

Williams82 ATTTGAGGAAAATATAGGAAAAAAATAGTTTGACTAAAGAAAAAATATACAAAAGTGAAA
Essex ............................................................
Forrest ...................................................G........

Williams82 TTATGTAATTTTAAAAGTATTGACCTGTTAATGTAACATTTTAAATAAATATAGTAAAAT
Essex ............................................................
Forrest ............................................................

Williams82 AATAGTTAATTAAAAAAAGAATCACAATAATATAAATAAATCATAATTTTTTAAAAATTC
Essex ............................................................
Forrest .............T..............................................

Williams82 AAATAAAATATTATTTGTTGAAAATTCAATCCGAATTCACACTTCAAGAAGGTTACCCGA
Essex ............................................................
Forrest ............................................................

Williams82 TATATCATCCTGTTATAAAAAAGTAAGAAACACGTTATAACACACATTTTCATGCACGTG
Essex ............................................................
Forrest ......................A.......................C.............

Williams82 AAAAATAAAAATAAAATATTTTTTTGATAAATATTAATTATTAATCTGTTAATTTTATTA
Essex ............................................................
Forrest ..........................G.................................

Williams82 ATATGAGAAATTAAACACATAATCTTTTACTTATTTATTTTTTCTTAACCCTCAATCAAT
Essex ............................................................
Forrest ............C...................G...........................

Williams82 CTTATATCCCTTATGATAAATCAAACACACTAATAAGTAATTCATTATTTGGTTAAATAC
Essex ............................................................
Forrest ............................................................

Williams82 TATCTCAAAATTTGTGCACAACAAGTTAAATGATAAACCTAACATGCACCGAATCACCCA
Essex ............................................................
Forrest ............................................................

Williams82 ACTATTGGTGTGTCTGTATTCCTGTTTAAGTACATTTATCGTTAATCCAAAGGCGAGAGA
Essex ............................................................
Forrest .......................C....................................

Williams82 GAACAGAAAGACGGAATGTTCTATTCACGTAAACATGCATAAAAAGATAACCCAAAAAAT
Essex ............................................................
Forrest ............................................................

Williams82 GTTGTTGGGTTTAATACATTTGTTTGATTATGAATAGTGTATTTGTATTAGTAATTTTAT
Essex ............................................................
Forrest ............................................................

Williams82 TAATTTTTGTCTGTGAAATTACTTTTGTACAAATAATATTTTTTTAAAAAAATTACAACA
Essex .............................................T..............
Forrest ...C.......................................AA...............

Williams82 AAAAAGTAGACAGAGAATAGAAAATATATAATAAAATAGATGATATAATAATAAAACTCT
Essex ............................................................
Forrest ..........A.................................................

Williams82 TAAATATAAGATGAAAAATAATTTATAATTAATTGAAGATTTAATAACTTTTACACTAAC
Essex ............................................................
Forrest ............................................................

Williams82 ATTCCACGTATATTAAACTTTTAAACAATAAGACTTGACTCAATTTGTTTTTGGTTTCAT
Essex ............................................................
Forrest ............................................................

Williams82 AGC---------------TAAAGTTTTTTAAACTACCAAACAATTAAAAGTTATCATTAA
Essex ...---------------..........................................
Forrest ...TATATATTATTAGTG..........................................

Williams82 TAATACTTTTAATTATTATAAAAATTAATAAATTTATCATATATAATGATATGTGATTGA
Essex ............................................................
Forrest ...C...........................................A............

Williams82 ATAATATTATAAAATTATTTTACACTAAAACTGATTTACCTAAATGTTAAAGAATTTTTT
Essex ............................................................
Forrest ............................................................

Williams82 AACTAGTAAAAAAAATATAAAAATTAACTCAAATGTATTTGCCAAATAAGTTGGGCCATT
Essex .....................................-......................
Forrest .............................T.......C-.....................

Williams82 TAATAACATTCTCTCTTATTTTCTTCTTGTTTTTAAAAGAATAAGTTGGACCAAATATAT
Essex ............................................................
Forrest .........................A.........C........................

Williams82 TTCTTAAAAAACTTTAAGAAAGGAATATAAAAATAATGCATGGAGATTATATA--TTTTT
Essex .....................................................--.....
Forrest ....................................................TTT.....

Williams82 AATAAATAATATGTGCATTTGATTGTATAAAGTTTTTCTATGTATTTTCATTCGGTCACA
Essex ............................................................
Forrest ............................................................

Williams82 ATTATTATATTTACTATCTTTGTTGATTTTATAACAATTACCTTAAAAATCTTATGAAAG
Essex ............................................................
Forrest ............G...............................................

Williams82 ATACTTTTTATAAGTTGACAATGTAAGTATGTAACTGATTTGAGTTTTTTAAAGTGTCAT
Essex ............................................................
Forrest ............................................................

Williams82 CGAAATATCAGTTGTTAATATTATCCTTTACCTTACATTTTTTAATTTTAAACTTGTATG
Essex ............................................................
Forrest ..........................C.................................

Williams82 ATTGACTAATTAAATGTCTAAAAACTTTAGATTTTTCATAATTTCATAATGAAATATTAA
Essex ............................................................
Forrest .....................................T......................

Williams82 TAACATTATATAATTTCAATTACTAAAATATTAATAGTTAACTCTTCCAGTTCTAGTTTA
Essex ............................................................
Forrest ............................................................

Williams82 ACTTCCCCAAACCTTTGTAGACTCTAGCTATTATTAAAAAAATGAAAAAACAAAAGGCAT
Essex ............................................................
Forrest ........................................................T...

Williams82 AACAAAACGCTGTAGCAGGAGTAGGATGTGGACCTAATTATTGTTGTATTGTAACGAAGC
Essex ............................................................
Forrest ............................................................

Williams82 TCAACGTTAGAGAGGCAGCTTTTGCTTTATAAAAACCAGAGATTGAACAACCAAAGAGC
Essex ...........................................................
Forrest ...........................................................

## *Glyma.10G017000* Gene Alignment

Since Forrest, PI 96354, and Lee 74 sequences are identical, only Forrest is shown in the following alignment. Likewise, as Essex and Bossier sequences are identical, only Essex is shown in the following alignment. CDS splice sites were validated by sequencing mRNA.

5’ UTR
CDS
3’ UTR

Williams82 TCAGTGTGGCAGTTTGGTCGTTGAGTGAGTGAGTGAGGCTGGTTTCACTTTCACATTGCC
Essex ............................................................
Forrest ............................................................

Williams82 TCTTCAAAATGACTTTCTCCTTTTCCTTCTTCACCATCACCACTCTGCTTTCTCTGTTCT
Essex ............................................................
Forrest ............................................................

Williams82 CTCTAATTCTGCTTCATGCCAATGCCTTCCCAGTCCCCATGCATCGCCACCCTCGCTTTG
Essex ............................................................
Forrest ........................................A...................

Williams82 CCACTCATAACTACAGAGATGCTCTCACTAAATCCATTCTCTTCTTTGAAGGCCAGAGGT
Essex ............................................................
Forrest ............................................................

Williams82 CAGGGAAGCTCCCTCCTAACCAGAGAATGTCTTGGAGGAGAGACTCTGGCCTCTCTGATG
Essex ............................................................
Forrest ............................................................

Williams82 GCTCAGCCATGCACGTATGCATATACCAACTCTCACATTACCATGTTTTCTGGTTTTTGT
Essex ............................................................
Forrest .................................................T.T........

Williams82 TTTTGGTTTGCTTATTTATGTGTGAATGTGCAATGCAGGTTGATTTAGTTGGAGGGTACT
Essex ............................................................
Forrest ..............................................G.............

Williams82 ATGATGCTGGGGACAATGTAAAATTTGGTTTTCCCATGGCCTTCACCACCACCATGCTTT
Essex ............................................................
Forrest ...................G............................T...........

Williams82 CATGGAGCGTTATTGAGTTTGGTGGGCTAATGAAAGGTGAGTTGCAGAATGCCAGAGAGG
Essex ............................................................
Forrest ............................................................

Williams82 CCATTCGCTGGGGCACTGATTATCTTCTCAAAGCCACTGCACATCCAAACACCATTTATG
Essex ............................................................
Forrest ............................................................

Williams82 TTCAGGTCAGTTAAGACAACAACACTCTTGCTAAAATCAACTTATACCCTTTTGAAAGTA
Essex ............................................................
Forrest ............................................................

Williams82 ACTATGGAAAAGTTTTTTTTTTCAAATATGGGGTAAGTCTTCTTTAGATTCTGATATTTG
Essex .................A..........................................
Forrest .................-..........................................

Williams82 TTTGTTTGTTTGTTATTTGGCTTTGCAGGTGGGAGACGCTAAGAAGGACCATGCTTGTTG
Essex ............................................................
Forrest ............................................................

Williams82 GGAGAGACCAGAGGACATGGACACACTAAGAAGCGTGTTTAAAATAGATGCAAACACACC
Essex ............................................................
Forrest ...................................C........................

Williams82 TGGTTCAGAAGTTGCCGCGGAAACTGCTGCAGCTCTTGCAGCTGCTTCTCTTGTTTTTAG
Essex ............................................................
Forrest ............................................................

Williams82 AAGAAGTGACCCCACATACTCCAAAGTTTTAGTGAGGAGAGCCATCAGGGTAAGTAAACC
Essex ............................................................
Forrest ............................................................

Williams82 TTTGGTTCAATTTTTGCTAATTATTATTTTTAATAGACCAAACTGCTCCCAACTAAATCT
Essex ............................................................
Forrest ............................................................

Williams82 TAAAAGAGTATTTTGTTTCTGAGTGTAGGTCTTCCAGTTTGCTGATAAGTACAGGGGATC
Essex ............................................................
Forrest C......C....................................................

Williams82 CTACAGCAATGCCTTGAAACCTTATGTGTGCCCCTTCTATTGCTCTTACTCTGGTTATCA
Essex ............................................................
Forrest ............................................................

Williams82 GGTAAAGTTTCAACTGTTCCATATGAGTGATGTTGTTCAGTTTAGTTGGATGAGACATAA
Essex ............................................................
Forrest ............................................................

Williams82 TCATTACTCAAATTCTTTTGATGAATAATTATTATGGTAGGATGAGCTGTTGTGGGGTGC
Essex ............................................................
Forrest ............................................................

Williams82 TGCCTGGCTGCACAAGGCTACCAGGAATCCAATGTACCTAAACTACATCAAAGTTAATGG
Essex ............................................................
Forrest ............................................................

Williams82 CCAGATCCTTGGGGCTGCAGAGTTTGACAACACCTTTGGGTGGGATAACAAGCATGCTGG
Essex ............................................................
Forrest ............................................................

Williams82 AGCAAGAATACTTCTTTCCAAGGTACAAATTACTCATCCATCACATGTATTCTGCTGTAT
Essex ............................................................
Forrest ............................................................

Williams82 ATATATTGCAAAACTAGATAGAAAGTAAAAACTAATAATGGTTTGAAATTTACATTTTTG
Essex ............................................................
Forrest ............................................................

Williams82 TGTTCCATGGTTTTAGGAATTCCTGGTTCAAAGGGTACAATCCCTCCATGACTACAAGGG
Essex ............................................................
Forrest ............................................................

Williams82 TCATGCGGACAATTTCGTCTGTTCTCTAATTCCTGGAACCTCTTTTTCTTCCACTCAATA
Essex ............................................................
Forrest ............................................................

Williams82 TACCCCAGGTGAAGTCCCTTTTCCTTCTTAATTCTTTTACTTATCCTCTCTCCCAGTGGA
Essex ............................................................
Forrest ............................................................

Williams82 AACTGCGCGTGTGTGTGGTCCAGTTTTTGTAACGGTTACTTGCTTGTTACTAGGTGGGCT
Essex ............................................................
Forrest ............................................................

Williams82 TCTTTTCAAGATGAGTGATAGCAACATGCAGTATGTCACATCCACCTCCTTCCTTCTGCT
Essex ............................................................
Forrest ......................................................A.....

Williams82 AACATATGCCAAATACTTGACCCAATCCCATATGCTTGTTAACTGTGGTGGAATCACAGT
Essex ............................................................
Forrest ............................................................

Williams82 AACCCCAAGGAGACTCCGGACAATAGCTAAGAAACAGGTTACAAAATGTGTCTATTATAT
Essex ............................................................
Forrest ............................................................

Williams82 GTTGAATAAGTTTTAATCAATTAAAATACTTTCTATATGATTAGTTAAAAATTATTTAAA
Essex ............................................................
Forrest ............................................................

Williams82 ATAATTAATTTTGATAAGTTTTATTTCTTAATCAACATAACTAAATCAGGAGCAGAAAAG
Essex ............................................................
Forrest ..............................T.............................

Williams82 AGAAAATTATTAGATGAGAAATAAAACAAACAAAAAAAAAATTCTAACCAATTATGAAGA
Essex ............................................................
Forrest ............................................................

Williams82 ACTTCTTAAACAAAGAATGTAAAAGAGTGCCACCAGAAAAAGTGTTGTTAGATTTCCTAT
Essex ............................................................
Forrest ......................G.....................................

Williams82 TCTTGTTAGTTGAACTTGA-TTTTTTTTTTAATTTTTGTAATTTTAGGTGGATTACTTGC
Essex ...................-........................................
Forrest ...................T........................................

Williams82 TTGGAGACAACCCGTTGAAGATGTCGTACATGGTGGGGTATGGTCCACGGTACCCACGAA
Essex ............................................................
Forrest ............................................................

Williams82 GGATACACCATAGGGGATCATCTCTACCGTCGATTGCTGTGCACCCGGGAAAGATCCAGT
Essex ............................................................
Forrest ............................................................

Williams82 GTTCCGCAGGGTTCAGTGTGATGAATTCACAATCTCCCAACCCAAACATTCTAGTGGGGG
Essex ............................................................
Forrest ............................................................

Williams82 CCATTGTTGGTGGACCGGATGAGCATGATAGGTTCCCAGATCAACGGTCAGATTATGAGC
Essex ............................................................
Forrest ............................................................

Williams82 AATCAGAGCCAGCTACATACATTAACTCACCCCTTGTAGGAGCACTGGCCTATCTTGCAC
Essex ............................................................
Forrest ............................................................

Williams82 ACTCATTCGGTCAACTCTAGGAACCAACCATATCGTGTATCCCATGTTTTATTTTACTAG
Essex ............................................................
Forrest ..........................................A.................

Williams82 TTTCGTTAGTTACTTACTTGTGTCTCCTAGAATTATGCACACACTCATTTGAGACCAGTA
Essex ............................................................
Forrest ............................................................

Williams82 CGGCAGTACCACACCACTAAGCCTTATAAACTTGCTTACAAGCTTAACTTTACTACTATG
Essex ............................................................
Forrest ............................................................

Williams82 TGTAACTTCATATTGCCGCCATGCTTTTGTAAGTATTATGATTTCGGAAAGTGTTATATA
Essex ............................................................
Forrest ............................................................

Williams82 TGTATCTCCCGTGATGTAGATGTGTTTGATTTTGCATGTTATTTGGGTGTAAAAAATGGA
Essex ............................................................
Forrest ............................................................

Williams82 TTAAGTTTGGAATAGCTAGGGTATCGGTTC
Essex ..............................
Forrest ..............................

## Glyma.10G017000 Protein Alignment

Williams82 MTFSFSFFTITTLLSLFSLILLHANAFPVPMHRHPRFATHNYRDALTKSILFFEGQRSGK
Forrest ..............................I.............................

Williams82 LPPNQRMSWRRDSGLSDGSAMHVDLVGGYYDAGDNVKFGFPMAFTTTMLSWSVIEFGGLM
Forrest .............................................I..............

Williams82 KGELQNAREAIRWGTDYLLKATAHPNTIYVQVGDAKKDHACWERPEDMDTLRSVFKIDAN
Forrest .....................................................A......

Williams82 TPGSEVAAETAAALAAASLVFRRSDPTYSKVLVRRAIRVFQFADKYRGSYSNALKPYVCP
Forrest ............................................................

Williams82 FYCSYSGYQDELLWGAAWLHKATRNPMYLNYIKVNGQILGAAEFDNTFGWDNKHAGARIL
Forrest ............................................................

Williams82 LSKEFLVQRVQSLHDYKGHADNFVCSLIPGTSFSSTQYTPGGLLFKMSDSNMQYVTSTSF
Forrest ............................................................

Williams82 LLLTYAKYLTQSHMLVNCGGITVTPRRLRTIAKKQVDYLLGDNPLKMSYMVGYGPRYPRR
Forrest ............................................................

Williams82 IHHRGSSLPSIAVHPGKIQCSAGFSVMNSQSPNPNILVGAIVGGPDEHDRFPDQRSDYEQ
Forrest ............................................................

Williams82 SEPATYINSPLVGALAYLAHSFGQL*
Forrest ..........................

## *Glyma.10G017000* Promoter and CDS Sequences

>Glyma.10G017000 Essex Promoter
GACAAGTAGCAAACTTTTAGGCAATTTTCCAATCAGAGAATATTATTGTCCATTGCAATTTGAAAAATAGAAACGGATTCAAGTAAAAACTTACATAATAACAACTAAAATAAAGAAGCCTAAGACAATGACCTTAATTGCTTCGAACTATCCACGGCCTTGGTGCGATGACACTAGAAATTTTCAAAACGAATTTAAATTGTTAAATTAACTTCAAAATTGAAAATATGAATTCATTAACTAAAAAACCAAAAAATATCTTTTATTCAATTACGATTTTCATATCAAAATTGAACCAAACCATATCATTTATAATATTATTATATTTTTATAAAAGTGACCTTGAAAAGAAATGTCATTTCATTATAATTAAGGATTTAAGGATGTTTATTTTTTAAAAGTTGTTCAAGGAATATAATATCAAAATCTTACCTTTATACTGAATAAACAAACTCATCCTTATAAACAACTACTTTCATATATTTAAGTAATGTAAAAAGTAAACTAATTAATTATCAAAGAAGCTAAATATTTGAGGAAAATATAGGAAAAAAATAGTTTGACTAAAGAAAAAATATACAAAAGTGAAATTATGTAATTTTAAAAGTATTGACCTGTTAATGTAACATTTTAAATAAATATAGTAAAATAATAGTTAATTAAAAAAAGAATCACAATAATATAAATAAATCATAATTTTTTAAAAATTCAAATAAAATATTATTTGTTGAAAATTCAATCCGAATTCACACTTCAAGAAGGTTACCCGATATATCATCCTGTTATAAAAAAGTAAGAAACACGTTATAACACACATTTTCATGCACGTGAAAAATAAAAATAAAATATTTTTTTGATAAATATTAATTATTAATCTGTTAATTTTATTAATATGAGAAATTAAACACATAATCTTTTACTTATTTATTTTTTCTTAACCCTCAATCAATCTTATATCCCTTATGATAAATCAAACACACTAATAAGTAATTCATTATTTGGTTAAATACTATCTCAAAATTTGTGCACAACAAGTTAAATGATAAACCTAACATGCACCGAATCACCCAACTATTGGTGTGTCTGTATTCCTGTTTAAGTACATTTATCGTTAATCCAAAGGCGAGAGAGAACAGAAAGACGGAATGTTCTATTCACGTAAACATGCATAAAAAGATAACCCAAAAAATGTTGTTGGGTTTAATACATTTGTTTGATTATGAATAGTGTATTTGTATTAGTAATTTTATTAATTTTTGTCTGTGAAATTACTTTTGTACAAATAATATTTTTTTTAAAAAATTACAACAAAAAAGTAGACAGAGAATAGAAAATATATAATAAAATAGATGATATAATAATAAAACTCTTAAATATAAGATGAAAAATAATTTATAATTAATTGAAGATTTAATAACTTTTACACTAACATTCCACGTATATTAAACTTTTAAACAATAAGACTTGACTCAATTTGTTTTTGGTTTCATAGCTAAAGTTTTTTAAACTACCAAACAATTAAAAGTTATCATTAATAATACTTTTAATTATTATAAAAATTAATAAATTTATCATATATAATGATATGTGATTGAATAATATTATAAAATTATTTTACACTAAAACTGATTTACCTAAATGTTAAAGAATTTTTTAACTAGTAAAAAAAATATAAAAATTAACTCAAATGTATTGCCAAATAAGTTGGGCCATTTAATAACATTCTCTCTTATTTTCTTCTTGTTTTTAAAAGAATAAGTTGGACCAAATATATTTCTTAAAAAACTTTAAGAAAGGAATATAAAAATAATGCATGGAGATTATATATTTTTAATAAATAATATGTGCATTTGATTGTATAAAGTTTTTCTATGTATTTTCATTCGGTCACAATTATTATATTTACTATCTTTGTTGATTTTATAACAATTACCTTAAAAATCTTATGAAAGATACTTTTTATAAGTTGACAATGTAAGTATGTAACTGATTTGAGTTTTTTAAAGTGTCATCGAAATATCAGTTGTTAATATTATCCTTTACCTTACATTTTTTAATTTTAAACTTGTATGATTGACTAATTAAATGTCTAAAAACTTTAGATTTTTCATAATTTCATAATGAAATATTAATAACATTATATAATTTCAATTACTAAAATATTAATAGTTAACTCTTCCAGTTCTAGTTTAACTTCCCCAAACCTTTGTAGACTCTAGCTATTATTAAAAAAATGAAAAAACAAAAGGCATAACAAAACGCTGTAGCAGGAGTAGGATGTGGACCTAATTATTGTTGTATTGTAACGAAGCTCAACGTTAGAGAGGCAGCTTTTGCTTTATAAAAACCAGAGATTGAACAACCAAAGAGC

>Glyma.10G017000 Essex CDS
ATGACTTTCTCCTTTTCCTTCTTCACCATCACCACTCTGCTTTCTCTGTTCTCTCTAATTCTGCTTCATGCCAATGCCTTCCCAGTCCCCATGCATCGCCACCCTCGCTTTGCCACTCATAACTACAGAGATGCTCTCACTAAATCCATTCTCTTCTTTGAAGGCCAGAGGTCAGGGAAGCTCCCTCCTAACCAGAGAATGTCTTGGAGGAGAGACTCTGGCCTCTCTGATGGCTCAGCCATGCACGTTGATTTAGTTGGAGGGTACTATGATGCTGGGGACAATGTAAAATTTGGTTTTCCCATGGCCTTCACCACCACCATGCTTTCATGGAGCGTTATTGAGTTTGGTGGGCTAATGAAAGGTGAGTTGCAGAATGCCAGAGAGGCCATTCGCTGGGGCACTGATTATCTTCTCAAAGCCACTGCACATCCAAACACCATTTATGTTCAGGTGGGAGACGCTAAGAAGGACCATGCTTGTTGGGAGAGACCAGAGGACATGGACACACTAAGAAGCGTGTTTAAAATAGATGCAAACACACCTGGTTCAGAAGTTGCCGCGGAAACTGCTGCAGCTCTTGCAGCTGCTTCTCTTGTTTTTAGAAGAAGTGACCCCACATACTCCAAAGTTTTAGTGAGGAGAGCCATCAGGGTCTTCCAGTTTGCTGATAAGTACAGGGGATCCTACAGCAATGCCTTGAAACCTTATGTGTGCCCCTTCTATTGCTCTTACTCTGGTTATCAGGATGAGCTGTTGTGGGGTGCTGCCTGGCTGCACAAGGCTACCAGGAATCCAATGTACCTAAACTACATCAAAGTTAATGGCCAGATCCTTGGGGCTGCAGAGTTTGACAACACCTTTGGGTGGGATAACAAGCATGCTGGAGCAAGAATACTTCTTTCCAAGGAATTCCTGGTTCAAAGGGTACAATCCCTCCATGACTACAAGGGTCATGCGGACAATTTCGTCTGTTCTCTAATTCCTGGAACCTCTTTTTCTTCCACTCAATATACCCCAGGTGGGCTTCTTTTCAAGATGAGTGATAGCAACATGCAGTATGTCACATCCACCTCCTTCCTTCTGCTAACATATGCCAAATACTTGACCCAATCCCATATGCTTGTTAACTGTGGTGGAATCACAGTAACCCCAAGGAGACTCCGGACAATAGCTAAGAAACAGGTGGATTACTTGCTTGGAGACAACCCGTTGAAGATGTCGTACATGGTGGGGTATGGTCCACGGTACCCACGAAGGATACACCATAGGGGATCATCTCTACCGTCGATTGCTGTGCACCCGGGAAAGATCCAGTGTTCCGCAGGGTTCAGTGTGATGAATTCACAATCTCCCAACCCAAACATTCTAGTGGGGGCCATTGTTGGTGGACCGGATGAGCATGATAGGTTCCCAGATCAACGGTCAGATTATGAGCAATCAGAGCCAGCTACATACATTAACTCACCCCTTGTAGGAGCACTGGCCTATCTTGCACACTCATTCGGTCAACTCTAG

>Glyma.10G017000 Forrest Promoter
GACAAGTAGCAAACTTTTAGGCAATTTTCCAATCATAGAATATTATTGTCCATTGCAAATTGAAAAATAGAAACGGATTCAAGTAAAAACTTACATAATAACAACTAAAATAAAATAAAATAAAGAAGCCTAAGACAATGACCTTAAATGCTTCGAACTATCCACGGCCTTGGTGCGATGACACTAGAAATTTTCAAAACGAATTTAAATTGTTAAATTAACTTCAAAATTGAAAATATGAATTCATTAACTAAAAAACCAAAAAATATCTTTTATTCAATTACGATTTTCATATCAAAATTGAACCAAACCATATCATTTATAATATTATTATATTTTTATAAAAGAAATGTCATTTCATTATAATTAAGGATTTAAGGATGTTTATTTTTTAAAAGTTGTTCAAGGAATATAATATCAAAATCTTACCTTTATACTGAATAAACAAACTCATCCTTATAAACAACTACTTTCATATATTTAAGTAATGTAAAAAGTAAACTAATTAATTATCAAAGAAGCTAAATATTTGAGGAAAATATAGGAAAAAAATAGTTTGACTAAAGAAAAAATATACAGAAGTGAAATTATGTAATTTTAAAAGTATTGACCTGTTAATGTAACATTTTAAATAAATATAGTAAAATAATAGTTAATTAATAAAAGAATCACAATAATATAAATAAATCATAATTTTTTAAAAATTCAAATAAAATATTATTTGTTGAAAATTCAATCCGAATTCACACTTCAAGAAGGTTACCCGATATATCATCCTGTTATAAAAAAATAAGAAACACGTTATAACACACACTTTCATGCACGTGAAAAATAAAAATAAAATATTTTTTTGGTAAATATTAATTATTAATCTGTTAATTTTATTAATATGAGAAATTCAACACATAATCTTTTACTTGTTTATTTTTTCTTAACCCTCAATCAATCTTATATCCCTTATGATAAATCAAACACACTAATAAGTAATTCATTATTTGGTTAAATACTATCTCAAAATTTGTGCACAACAAGTTAAATGATAAACCTAACATGCACCGAATCACCCAACTATTGGTGTGTCTGTATTCCTCTTTAAGTACATTTATCGTTAATCCAAAGGCGAGAGAGAACAGAAAGACGGAATGTTCTATTCACGTAAACATGCATAAAAAGATAACCCAAAAAATGTTGTTGGGTTTAATACATTTGTTTGATTATGAATAGTGTATTTGTATTAGTAATTTTATTAACTTTTGTCTGTGAAATTACTTTTGTACAAATAATATTTTTAAAAAAAAATTACAACAAAAAAGTAGAAAGAGAATAGAAAATATATAATAAAATAGATGATATAATAATAAAACTCTTAAATATAAGATGAAAAATAATTTATAATTAATTGAAGATTTAATAACTTTTACACTAACATTCCACGTATATTAAACTTTTAAACAATAAGACTTGACTCAATTTGTTTTTGGTTTCATAGCTATATATTATTAGTGTAAAGTTTTTTAAACTACCAAACAATTAAAAGTTATCATTAATAACACTTTTAATTATTATAAAAATTAATAAATTTATCATATATAATAATATGTGATTGAATAATATTATAAAATTATTTTACACTAAAACTGATTTACCTAAATGTTAAAGAATTTTTTAACTAGTAAAAAAAATATAAAAATTAACTTAAATGTACTGCCAAATAAGTTGGGCCATTTAATAACATTCTCTCTTATTTTCTTATTGTTTTTACAAGAATAAGTTGGACCAAATATATTTCTTAAAAAACTTTAAGAAAGGAATATAAAAATAATGCATGGAGATTATATTTTTTTTTAATAAATAATATGTGCATTTGATTGTATAAAGTTTTTCTATGTATTTTCATTCGGTCACAATTATTATATTTGCTATCTTTGTTGATTTTATAACAATTACCTTAAAAATCTTATGAAAGATACTTTTTATAAGTTGACAATGTAAGTATGTAACTGATTTGAGTTTTTTAAAGTGTCATCGAAATATCAGTTGTTAATATTATCCCTTACCTTACATTTTTTAATTTTAAACTTGTATGATTGACTAATTAAATGTCTAAAAACTTTAGATTTTTCTTAATTTCATAATGAAATATTAATAACATTATATAATTTCAATTACTAAAATATTAATAGTTAACTCTTCCAGTTCTAGTTTAACTTCCCCAAACCTTTGTAGACTCTAGCTATTATTAAAAAAATGAAAAAACAAAAGTCATAACAAAACGCTGTAGCAGGAGTAGGATGTGGACCTAATTATTGTTGTATTGTAACGAAGCTCAACGTTAGAGAGGCAGCTTTTGCTTTATAAAAACCAGAGATTGAACAACCAAAGAGC

>Glyma.10G017000 Forrest CDS
ATGACTTTCTCCTTTTCCTTCTTCACCATCACCACTCTGCTTTCTCTGTTCTCTCTAATTCTGCTTCATGCCAATGCCTTCCCAGTCCCCATACATCGCCACCCTCGCTTTGCCACTCATAACTACAGAGATGCTCTCACTAAATCCATTCTCTTCTTTGAAGGCCAGAGGTCAGGGAAGCTCCCTCCTAACCAGAGAATGTCTTGGAGGAGAGACTCTGGCCTCTCTGATGGCTCAGCCATGCACGTTGATTTGGTTGGAGGGTACTATGATGCTGGGGACAATGTGAAATTTGGTTTTCCCATGGCCTTCACCATCACCATGCTTTCATGGAGCGTTATTGAGTTTGGTGGGCTAATGAAAGGTGAGTTGCAGAATGCCAGAGAGGCCATTCGCTGGGGCACTGATTATCTTCTCAAAGCCACTGCACATCCAAACACCATTTATGTTCAGGTGGGAGACGCTAAGAAGGACCATGCTTGTTGGGAGAGACCAGAGGACATGGACACACTAAGAAGCGCGTTTAAAATAGATGCAAACACACCTGGTTCAGAAGTTGCCGCGGAAACTGCTGCAGCTCTTGCAGCTGCTTCTCTTGTTTTTAGAAGAAGTGACCCCACATACTCCAAAGTTTTAGTGAGGAGAGCCATCAGGGTCTTCCAGTTTGCTGATAAGTACAGGGGATCCTACAGCAATGCCTTGAAACCTTATGTGTGCCCCTTCTATTGCTCTTACTCTGGTTATCAGGATGAGCTGTTGTGGGGTGCTGCCTGGCTGCACAAGGCTACCAGGAATCCAATGTACCTAAACTACATCAAAGTTAATGGCCAGATCCTTGGGGCTGCAGAGTTTGACAACACCTTTGGGTGGGATAACAAGCATGCTGGAGCAAGAATACTTCTTTCCAAGGAATTCCTGGTTCAAAGGGTACAATCCCTCCATGACTACAAGGGTCATGCGGACAATTTCGTCTGTTCTCTAATTCCTGGAACCTCTTTTTCTTCCACTCAATATACCCCAGGTGGGCTTCTTTTCAAGATGAGTGATAGCAACATGCAGTATGTCACATCCACCTCCTTCCTACTGCTAACATATGCCAAATACTTGACCCAATCCCATATGCTTGTTAACTGTGGTGGAATCACAGTAACCCCAAGGAGACTCCGGACAATAGCTAAGAAACAGGTGGATTACTTGCTTGGAGACAACCCGTTGAAGATGTCGTACATGGTGGGGTATGGTCCACGGTACCCACGAAGGATACACCATAGGGGATCATCTCTACCGTCGATTGCTGTGCACCCGGGAAAGATCCAGTGTTCCGCAGGGTTCAGTGTGATGAATTCACAATCTCCCAACCCAAACATTCTAGTGGGGGCCATTGTTGGTGGACCGGATGAGCATGATAGGTTCCCAGATCAACGGTCAGATTATGAGCAATCAGAGCCAGCTACATACATTAACTCACCCCTTGTAGGAGCACTGGCCTATCTTGCACACTCATTCGGTCAACTCTAG

## *Glyma.10G017100* Promoter Alignment

Williams82 GCACCCAACCCTTTATGAACAAAGCCAATGTGACATTGTGAAGCTTCAAGTCATCAGAGA
Bossier ............................................................
Essex ............................................................
Forrest ............................................................
PI96354 ............................................................

Williams82 AAGATGAGGACAACTCATTCTTCGCTCTTGCGTCTGAACTCTGTAACCCATTTAAACATG
Bossier ............................................................
Essex ............................................................
Forrest ............................................................
PI96354 ............................................................

Williams82 TTTGTTGGTTAGTCAAAACGGCACTGAGATAGGTTTCAAAATCTTTAGCTTCAGAAGTGT
Bossier ............................................................
Essex ............................................................
Forrest .....-..............................................T.......
PI96354 .....-................G.............................T.......

Williams82 GAAGAAAACCACTAACTTGGTTAGCAGTGGCATAGATGTTTGATAAGTATTCTAGACTTT
Bossier ............................................................
Essex ............................................................
Forrest .................C..........................................
PI96354 .................C..........................................

Williams82 GTTCAACAACGAATTGCCAATCTTCAAGAGCACCAAGTGAGTATTGAGACAAAGACAAGC
Bossier ............................................................
Essex ............................................................
Forrest ............................................................
PI96354 ............................................................

Williams82 TACCTTGAAGATATGAGTCCACTAAGTTCAAGAACTTACGAGCTTGGGACAAGGACTTTC
Bossier ............................................................
Essex ............................................................
Forrest ............................................................
PI96354 ............................................................

Williams82 GAAAAGAAATACGACCATAGTCGAAGATGTTTCCATTTTGATTAGCAAGCATGGTTTTGC
Bossier ............................................................
Essex ............................................................
Forrest ............................................................
PI96354 ............................................................

Williams82 AATAAGTAGGGTCTAGGGTGGACTCACAAATAGTTTCGGGTGGAACAATGGAGGATAAAC
Bossier ............................................................
Essex ............................................................
Forrest ............................................................
PI96354 ............................................................

Williams82 TGGTATATGCTATGGACAATGAGGCCAAGACCGAGAAGGAAAGCATTTAGGAAACAAACA
Bossier ............................................................
Essex ............................................................
Forrest .......................A..............................A.....
PI96354 .......................A..............................A.....

Williams82 ACTTAAGGTCAAAGATATACTTGACAGTCATTGGTTTT---TTTT-TTT-----------
Bossier ......................................---....-...T----------
Essex ......................................---....-...T----------
Forrest ................................A.....CTC....G...TTCTTCGTATG
PI96354 ................................A.....CTC....G...TTCTTCGTATG

Williams82 -------------------------------------------------------T----
Bossier ------------------------------------------------------------
Essex ------------------------------------------------------------
Forrest CTTGTAAGGAACCCATGAGTTGCTCAATAGTCATGGTCTTTAAATCCTTGTTTTC.TCAA
PI96354 CTTGTAAGGAACCCATGAGTTGCTCAATAGTCATGGTCTTTAAATCCTTGTTTTC.TCAA

Williams82 -----------ATGAAG----------GATTT----------------------------
Bossier -----------......----------.....----------------------------
Essex -----------......----------.....----------------------------
Forrest TGTTGGTAACA......TCAAAACTTG.....AAAGTTCGAAGTATTTTTTCCATGACCT
PI96354 TGTTGGTAACA......TCAAAACTTG.....AAAGTTCGAAGTATTTTTTCCATGACCT

Williams82 --A---------------------------------------------------------
Bossier --.---------------------------------------------------------
Essex --.---------------------------------------------------------
Forrest TC.CCTCATCAACATTTTCACCATTTCTTTTAAGTTGATTGACTACGGCCAATACTCGAG
PI96354 TC.CCTCATCAACATTTTCACCATTTCTTTTAAGTTGATTGACTACGGCCAATACTCGAG

Williams82 ----------------------GG------------------------------------
Bossier ----------------------..------------------------------------
Essex ----------------------..------------------------------------
Forrest AAAAATAATCAGAAATTGACTC..ACTCCTCCATAAACAAACGCTCAAAGTCACCTCTAA
PI96354 AAAAATAATCAGAAATTGACTC..ACTCCTCCATAAACAAACGCTCAAAGTCACCTCTAA

Williams82 --GT-----------T--------------------------------------------
Bossier --..-------------T------------------------------------------
Essex --..-------------T------------------------------------------
Forrest GA..TTGAAGACGAA.CTTTTTTACCTGCTCAACTCCTTTGTTGCAAGTTTGAAGCTTAT
PI96354 GA..TTGAAGACGAA.CTTTTTTACCTGCTCAACTCCTTTGTTGCAAGTTTGAAGCTTAT

Williams82 ------------------------------------------------------------
Bossier ------------------------------------------------------------
Essex ------------------------------------------------------------
Forrest CCCATGCTTCTTTGGCCGTCGTTGCGTTGGATATCTTCTCAAATGTATCTTCATCCACCG
PI96354 CCCATGCTTCTTTGGCCGTCGTTGCGTTGGATATCTTCTCAAATGTATCTTCATCCACCG

Williams82 ------------------------------------------------------------
Bossier ------------------------------------------------------------
Essex ------------------------------------------------------------
Forrest ATTGATAAATGAGAAAGAGAGCTTTCTTGTCTCTCTTTCTTGACTCCTTCAACGTCTCCT
PI96354 ATTGATAAATGAGAAAGAGAGCTTTCTTGTCTCTCTTTCTTGACTCCTTCAACGTCTCCT

Williams82 ------------------------------------------------------------
Bossier ------------------------------------------------------------
Essex ------------------------------------------------------------
Forrest TTACACCTTGGCTTAGCGAGACTTCATCTTGCTCCTCGAAGCCATTCTCTACGATATCCC
PI96354 TTACACCTTGGCTTAGCGAGACTTCATCTTGCTCCTCGAAGCCATTCTCTACGATATCCC

Williams82 ----------------------------------------------------GTTGTT--
Bossier ----------------------------------------------------......--
Essex ----------------------------------------------------......--
Forrest ACACATCTTGAGCTCCTAGTAGCGCCTTCATCTTGATACTCCAATTATCATA......CT
PI96354 ACACATCTTGAGCTCCTAGTAGCGCCTTCATCTTGATACTCCAATTATCATA......CT

Williams82 ------------------------------------------------------------
Bossier ------------------------------------------------------------
Essex ------------------------------------------------------------
Forrest TTGTGAGCATCGGCATTTGGAAATGAAAACCTCCATTCGCCATCTTTTGAGGATCTTGAA
PI96354 TTGTGAGCATCGGCATTTGGAAATGAAAACCTCCATTCGCCATCTTTTGAGGATCTTGAA

Williams82 ------------------------------------------------------------
Bossier ------------------------------------------------------------
Essex ------------------------------------------------------------
Forrest GCTCTGATACCACTTTGTTGGAAATAAGGCTTTTTATGTTTAGGAAAAGTGTTTAGGAAT
PI96354 GCTCTGATACCACTTTGTTGGAAATAAGGCTTTTTATGTTTAGGAAAAGTGTTTAGGAAT

Williams82 ------------------------------------------------------------
Bossier ------------------------------------------------------------
Essex ------------------------------------------------------------
Forrest ATTGGAGACTTTGAATAGAAACTTGATAGGAAGGAGAATTCTTTATGGAGGAGAGAACTT
PI96354 ATTGGAGACTTTGAATAGAAACTTGATAGGAAGGAGAATTCTTTATGGAGGAGAGAACTT

Williams82 ----------------------------------------------ACTAC-----GGA-
Bossier ----------------------------------------------.....-----...-
Essex ----------------------------------------------.....-----...-
Forrest TGTATTTTTGCTTGATACAAATGTGTAGGATTACATCTCTATTTAT.....TCTAA...G
PI96354 TGTATTTTTGCTTGATACAAATGTGTAGGATTACATCTCTATTTAT.....TCTAA...G

Williams82 ------------------------------------------------------------
Bossier ------------------------------------------------------------
Essex ------------------------------------------------------------
Forrest AACTCTAGACACACTAATTCTAGAGAGTTCTCAACTCTAGAGATCCAAAGAGTATTCTAG
PI96354 AACTCTAGACACACTAATTCTAGAGAGTTCTCAACTCTAGAGATCCAAAGAGTATTCTAG

Williams82 ------------------------------------------------------------
Bossier ------------------------------------------------------------
Essex ------------------------------------------------------------
Forrest AGAATATTAAAACCATAAGAAATATCTAGACATTCCAAACACTACAAGAATTCTCTAGAA
PI96354 AGAATATTAAAACCATAAGAAATATCTAGACATTCCAAACACTACAAGAATTCTCTAGAA

Williams82 ---TGA------------------------------------------------------
Bossier ---...------------------------------------------------------
Essex ---...------------------------------------------------------
Forrest ACA...CCCATAATTACTTAAGCCCAAAATAACTAAGTCCAAGAAACCAAATAATTAATT
PI96354 ACA...CCCATAATTACTTAAGCCCAAAATAACTAAGTCCAAGAAACCAAATAATTAATT

Williams82 ----------------------------ACAAATAAGGTGCATGACTAGTTTGTCTTATA
Bossier ----------------------------................................
Essex ----------------------------................................
Forrest TGGGCCCAAATCAAGTTTATATTTCAAC................................
PI96354 TGGGCCCAAATCAAGTTTATATTTCAAC................................

Williams82 TAGCGGACGAAGATTTTGTGTTATTATAAGGATTAGTGGGAATGAACTATGGGAGCTTTG
Bossier ............................................................
Essex ............................................................
Forrest ............................................................
PI96354 ............................................................

Williams82 TCAGTTAGAAGTCAACATCCAAAAGGAATTTGTCTTGGTGTGTTTGAAGTTGTTGCCATG
Bossier ............................................................
Essex ............................................................
Forrest .....................................-.T....................
PI96354 .....................................-.T....................

Williams82 GCTACCACGAATGCAGTCCCAACCGGCCATTTTGAAATTTTTTATTAGGAGCTTAGGAGG
Bossier ............................................................
Essex ............................................................
Forrest ............................................................
PI96354 ............................................................

Williams82 AACATACATTAAGTTTACTATAAACGTCCATGTATGCAAATATTCCTTGGGATTATATTT
Bossier ............................................................
Essex ............................................................
Forrest ............................................................
PI96354 ............................................................

Williams82 ATTCGTTTGAATTGATATTAAGGTTTGTTTTGGTACATGGTTTATTACCTTGTGACATAT
Bossier ............................................................
Essex ............................................................
Forrest ............................................................
PI96354 ............................................................

Williams82 ATTTAATTTAATTTTAGTTTGTCAACGCGGGATTGGTATCCAACAAAAAAACATGTATGT
Bossier ............................................................
Essex ............................................................
Forrest ............................................................
PI96354 ............................................................

Williams82 ATATATTGCTGGTCATGCTTCTATTTCTTATATTAATAATATTTCTCTTGGATATTGTAT
Bossier ............................................................
Essex ............................................................
Forrest ............................................................
PI96354 ............................................................

Williams82 TGGCTCAGGAGGCCCTGCCCCGCTATTGAGGAAATTAAGATATATTCAGAATATTATCAA
Bossier ............................................................
Essex ............................................................
Forrest ............................................................
PI96354 ............................................................

Williams82 GTCAGACGTAACACATAGTCACATAACTGAAATTGAAGATCAAATATTCATATATGCTTT
Bossier ............................................................
Essex ............................................................
Forrest ............................................................
PI96354 ............................................................

Williams82 GGGAGCAAAGCTTTTAGATTTTTATGTTACTTGATTTTACACATCAATTTCACATTTCAG
Bossier ............................................................
Essex ............................................................
Forrest ............................................................
PI96354 ............................................................

Williams82 GTTAATTTTGTATATATCTTTCTATAAAACTAGCCCTTCCAAAAGAACAAGAAATAAACT
Bossier ............................................................
Essex ............................................................
Forrest ............................................................
PI96354 ............................................................

Williams82 GCAAAAGGAATTACCATTTCAGCCAAACAGAATTAGCCTGAAGCAATATATTTAAAGCTT
Bossier ............................................................
Essex ............................................................
Forrest ............................................................
PI96354 ............................................................

Williams82 TCCTACCAGAAAGGCAGGTATGAAATAATTAAGAGAGTGGAAAAGGGAATTGAAGCTCCA
Bossier ............................................................
Essex ............................................................
Forrest ............................................................
PI96354 ............................................................

Williams82 GTACTTGATGCAGGAGCTAAAATGATTAAGGATCTCACGCATAACAACACGTTGCATACT
Bossier ............................................................
Essex ............................................................
Forrest ............................................................
PI96354 ............................................................

Williams82 CGAGAGTTCAATTAATCGAGGTGGCATATTTAACCTAGTCAGTATGAGGTCCATGTCACA
Bossier ............................................................
Essex ............................................................
Forrest ............................................................
PI96354 ............................................................

Williams82 TTATGACTAAAGCTAGCTCATTTTTTTTTCAAAAAATAAAAATAAAAATAAAAAAATAAA
Bossier ............................................................
Essex ............................................................
Forrest ...........................-........-------------........---
PI96354 ...........................-........-----------------....---

Williams82 AAATAAAATATATATATATATATATATATATATATATATATATATATATATATATATAGT
Bossier ....................................................------..
Essex ....................--------------------------------------..
Forrest -------.................................------------------..
PI96354 -------.....................................--------------..

Williams82 TTCACAGTGTCAACGTGAAACACCCCACTCCATGTTACAAT
Bossier .........................................
Essex .........................................
Forrest .........................................
PI96354 .........................................

## *Glyma.10G017100* Gene Alignment

As Essex, Bossier, and Williams82 sequences are identical, only Williams82 is shown in the following alignment. CDS splice sites were validated by sequencing mRNA.

5’ UTR
CDS
3’ UTR

Williams82 CCAACCACTCTATTTATCCACAGCACGTACCCTGCTAGCTAGTTACACATACACAGTAAT
Forrest ............................................................
PI96354 ............................................................

Williams82 CCTAACCCACCTCCTAAGTCCTATCACATCATGAACAACCTCACACTTGCATCCATTCTG
Forrest ............................................................
PI96354 ............................................................

Williams82 ACCGTGATTTCTTCTCTACTATTCTTTGGAACAACTCATTTAACCAACACCCAAACAACA
Forrest ............................................................
PI96354 ............................................................

Williams82 AGAGTCCCAGATCAAAAACATAAACACCTCCATTTCCAAAAGCACATACAAGTAGTAGCC
Forrest ............................................T...............
PI96354 ............................................T...............

Williams82 CAATCCACATGCGAAGGAACACTCTACCCAGACCTATGTGTCTTAACACTAGCCACATTC
Forrest ............................................................
PI96354 ............................................................

Williams82 CCAGATCTCACAACAAAATCTGTCCCACAAGTGATATCCTCAGTGGTCAACCATACCATG
Forrest ............................................................
PI96354 ............................................................

Williams82 TACGAGGTAAGATCAACGTCCTACAACTGCAGCGGCCTCAAAAAGATGCTCAAAAACCTC
Forrest ......A.....................................................
PI96354 ......A.....................................................

Williams82 AACCCACTCGACCAGAGAGCCCTCGACGACTGTCTCAAACTGTTTGAAGACACCAGCGTC
Forrest ...........................T................................
PI96354 ...........................T................................

Williams82 GAGCTCAAAGCCACCATCGACGATCTCTCCATCAAGAGCACCATAGGGTCTAAACTGCAC
Forrest ..................................................G....G...T
PI96354 ..................................................G....G...T

Williams82 CATGACTTGCAGACTCTGCTGAGTGGAGCAATGACCAACTTGTACACGTGCCTCGATGGC
Forrest .................................................A..........
PI96354 .................................................A..........

Williams82 TTTGCGTACAGCAAAGGGCGCGTGGGGGACAGAATCGAGAAGAAGCTGCTTCAAATATCG
Forrest ............................................................
PI96354 ............................................................

Williams82 CATCACGTGAGCAACTCGTTGGCCATGCTGAACAAAGTGCCTGGAGTTGAGAAATTAACA
Forrest ............................................................
PI96354 ............................................................

Williams82 ACTTCTTCGGAATCTGATGAGGTGTTTCCAGAATATGGAAAGATGCAAAAGGGGTTCCCT
Forrest ...........T................................................
PI96354 ...........T................................................

Williams82 TCGTGGGTGTCCTCTAAAGACCGAAAGCTTCTTCAAGCTAAAGTGAATGAGACCAAGTTC
Forrest ..............C..................T..........................
PI96354 ..............C..................T..........................

Williams82 AATCTTGTTGTTGCCAAAGATGGCACTGGCAACTTCACCACTATAGGGGAAGCACTGTCT
Forrest ............................................................
PI96354 ............................................................

Williams82 GTGGCTCCCAACTCAAGCACAACTAGGTACATAACAAAATACTTTATTTCAGACCAATGT
Forrest ............................................................
PI96354 ............................................................

Williams82 GATTGATTATAATTTACACTAGTATAAATGAATTACATAGTAAAAACGTAGTACCAATGG
Forrest .................T..........................................
PI96354 .................T..........................................

Williams82 GATACAAATTAAGAGAAAAGAGAAAGGTGTTAGTAGAGTGTCAATTTTTTTTTAAAGATT
Forrest ............................................................
PI96354 ............................................................

Williams82 TTTAATAATGCCATGTATTCAATTGGAAATGTATACACTATATACAGTGAGCTTTTTTTT
Forrest ............................................................
PI96354 ............................................................

Williams82 TTTTT----ATGTATAAGTGAAAAGAATAAATATTAAGCATTAGTCAAAATAAAATACAG
Forrest .....TTTT...................................................
PI96354 .....TTTT...................................................

Williams82 AAGTCATATGAGTTATTTAAAAATCACGTTAAGAAACTCATTCTCACATTATATCTATGT
Forrest ............................................................
PI96354 ............................................................

Williams82 ATAATTATATATGACTTAGATGTACTGTTTTCAATCTGATGTAAGAAACTCATTCTTACA
Forrest ....................................C.......................
PI96354 ....................................C.......................

Williams82 TTATACCTCTCCTATTTGATTGTAAGAAATATTTCTTCATTGGGTTGTAAAATTAAATGA
Forrest ............................................................
PI96354 ............................................................

Williams82 GTGTAAGTGATAACAGGTTTGTGATACACGTAACGGCGGGGGCATACTTCGAGAATGTGG
Forrest ............................................................
PI96354 ............................................................

Williams82 AAGTGATAAGGAAGAAGACGAATCTAATGTTTGTTGGAGACGGTATTGGAAAGACAGTAG
Forrest ............................................................
PI96354 ............................................................

Williams82 TGAAAGGCAGTAGGAATGTTGAGGACGGGTGGACCATTTTTCAATCTGCTACTGTTGGTA
Forrest ..................................A.........................
PI96354 ..................................A.........................

Williams82 AGTATTAAATTTAGAGTGCAGCAATGCATTCGGAAGGTCCCCTTAAAAATCGGTTCGTCA
Forrest .....-------------------------------------------------------
PI96354 .....-------------------------------------------------------

Williams82 GCATTCATCGGGCAGAATATGGCTCTGATACCATATTAAATTTATAGTGTAGCAAAGACA
Forrest ------------------------------------------------------------
PI96354 ------------------------------------------------------------

Williams82 TTCGAAAAATTCCCTTAAAAACCGGTTTATAAGGGGTGGCCTACCGAACTATATAAGTAC
Forrest ------------------------------------------------------------
PI96354 ------------------------------------------------------------

Williams82 TTATCAAAATTTGCTAAACATCCAATGTAGGACTATTTTCAACAGTAAATATGTCTTTCT
Forrest ----------------------------------------------------........
PI96354 ----------------------------------------------------........

Williams82 TCTTCCCACCTTCTGCTACTATTTTTAATTAGTTATGTTTTTTTCTTGGTTTGATATTCC
Forrest ............................................................
PI96354 ............................................................

Williams82 AACTTTGAAAAATAAAAATAGGTATGATATATAGAATGGTCATAAGTGAGATTTGATTAG
Forrest ............................................................
PI96354 ............................................................

Williams82 TAGCTCGGTCACTTAATAAAGTTTTATAAATGCTCTACTTTGAGGCTGGCGTTGTGATTG
Forrest ............................................................
PI96354 ............................................................

Williams82 TTAAGTCAAAGACAGAGAAAAAAAATCATTAGAAAAAGTTAGAACAAGACAACGAAAATG
Forrest ...............--...........................................
PI96354 ...............--...........................................

Williams82 GCAACTTGATGCACAGCCCACATGTTGGTTTAAAAATTATTAATAAACTAATTTTGCTAG
Forrest ............................................................
PI96354 ............................................................

Williams82 GTGATTTTGTAGTTAAAATACATAAAGTAGATGTGGGAAGATCATTTTAAATGGTTTGGA
Forrest .........................T..................................
PI96354 .........................T..................................

Williams82 CAGACATGGACTACTTGGGTTTGTCAAGACATCGTGCAGTTCCTGCATTGCATTGAAAAA
Forrest ......C.....-...............................................
PI96354 ......C.....-...............................................

Williams82 TAGCGTTGTAGAGGTTGATGCATGTAATTATCTTTTGTTTTGTTTTGTGTGTGTACACTA
Forrest ...T...........................T....-.......................
PI96354 ...T...........................T....-.......................

Williams82 GTAAAATCCTACCTCTTGTGAGAATAAGCACAACTTAGTTTTGAATGTTTGGAATAAAAC
Forrest ................G...........................................
PI96354 ................G...........................................

Williams82 CAAGGAAAGTTCAAAAGATAAAAGGCTTTGTCATTTTAAAATTTTTGAGGATTCTGCTTT
Forrest ..............................C.........------..------------
PI96354 ..............................C.........------..------------

Williams82 CCGTGATTGAACATAATGCTGAAAAAAGATAGGTGATGAGTGCATGTATATATGTTTAAA
Forrest ------------------.----------............A..................
PI96354 ------------------.----------............A..................

Williams82 GACTATTTTCAAAGGCATCACTGCAAATTTAAACTACCCCAGCTATCAATGACGTATGCA
Forrest ............................................................
PI96354 ............................................................

Williams82 TGAAATATAAATAGTGTCAAAGTGATGCTAGTATTATTGGTTACAATGCTGACTAGCTAG
Forrest ............................................................
PI96354 ............................................................

Williams82 ATTCCAAAATGCTTGTTTTTTTTTTGTGATCATTGCCCGTCGTCAACTATTCCTGTTATG
Forrest ...........G.............T.-............T...................
PI96354 ...........G.............T.-............T...................

Williams82 GGAATAAATTTTGAAGACTCTAATTTTGTGTTTTCAGTGTTCTGTTTGGTTTTTCCAGTT
Forrest ..............G.............................................
PI96354 ..............G.............................................

Williams82 ATATTGCAATCCAGTAATATCAATTGGACTTCTCCATCAGACAACGGGTCTCAATCACAA
Forrest ..............................................C.............
PI96354 ..............................................C.............

Williams82 TGACATTAGCAAATATGTATTGTAAATAAATCAAATTTCTTATTTCTTGTTAAAGGTAGC
Forrest ................A......................................T....
PI96354 ................A......................................T....

Williams82 AGTAATAAACCTAGATTTGGGATCTCCTGCCCGAAAGCATGTTTTCTTTATTAATATTTT
Forrest ............................................................
PI96354 ............................................................

Williams82 CCGAGCTATGAGTGGTGACACCATTTTTTGGAAAGAAAATGACGTGAGTAATCTTGGTGA
Forrest ............................................................
PI96354 ............................................................

Williams82 AAGATAGAAAAAGAACAGAGAAAAAAAACATAACCGGTGTGGTCTAATGAGAAGAAATAA
Forrest ............................................................
PI96354 ............................................................

Williams82 AAATAGAATTGGAAACTCTTTTATAAGACAAAAAAAAAAAAAAATACATGCAGGCAG---
Forrest ..........A..................C...........................GCA
PI96354 ..........A..................C...........................GCA

Williams82 ----AGGTAGGACTATCTTTGGCCTTAGATACATGATAATTTTATTTATATTTTCGCGAA
Forrest GAGG..............C.........................................
PI96354 GAGG..............C.........................................

Williams82 AAGTAGAGTTACCTCTATGGTTGGTCAAAGTAAAGGGTCCACATGCCTATCCACGCCCAC
Forrest ............................................................
PI96354 ............................................................

Williams82 CTAAAGATCTGCATAATTCTCATCACAACCAAACTACTTTTTCAGTGCCATTAGGTCCAC
Forrest ............................................................
PI96354 ............................................................

Williams82 TTTGCTTGTTTTTTCTTCTATGCTTCCCCTTTTACTTTTCTGCTTTTGCTCTTAACAAAT
Forrest ..................................................T.........
PI96354 ..................................................T.........

Williams82 AAAAATGCTTTAATTTCCTCTTTTGGGATAACATGGATAAAAGAAAGTGAATTTTATGAT
Forrest ............................................................
PI96354 ............................................................

Williams82 CTTTATTATTATTCATGTGACAAATAATGTGCAGTACAAAGTTAACAGACTTTTAGTTCT
Forrest .........---..............................................T.
PI96354 .........---..............................................T.

Williams82 CTTTCTTCGTTTCAATGATACCTTTATGTTGAGTTTGTGTCTCCCAGCAAAGTTTGCTCT
Forrest ...........................................T................
PI96354 ...........................................T................

Williams82 TTATTGTTCATTTATGAATAATGATCCAAAATCTAATCCTAACAGCGTTGACAATAATGG
Forrest ............................................................
PI96354 ............................................................

Williams82 TATGTTGCAACTATTCAGTTGTAACTCAGTTGGCAACACATCTTAAGTTTATGAAAAAAA
Forrest ......................................G..............G......
PI96354 ......................................G..............G......

Williams82 GATCTGTGTTCAATCCTTACAAGAAAAAGGATGAATAATTTTAAATGTAATTAAGTCTCG
Forrest A........................................A..................
PI96354 A........................................A..................

Williams82 AACAGAATGATTATGATTGTTGTTAATAAAGGAAAATGGTATGTTGCAGCTGTTGTAGGA
Forrest ..............................A.............................
PI96354 ..............................A.............................

Williams82 GCAGGATTCATAGCAAAGGGTATAACATTTGAGAAGTCAGCAGGACCCGACAAACACCAA
Forrest ............................................................
PI96354 ............................................................

Williams82 GCTGTGGCACTGAGAAGTGGTGTGCTGACTTCTCAGCTTTCTACCAATGCAGTTTCGTTG
Forrest ............................................................
PI96354 ............................................................

Williams82 GCTACCAGGACACTCTCTACGTCCATTCCCTGCGCCAATTCTACCGTGAACGTGACATTT
Forrest ..................................T.........................
PI96354 ..................................TT........................

Williams82 ATGGCACTGTAGACTTCATTTTCGGCAATGCAGCTGTGGTATTCCAAAACTGCAACTTAT
Forrest ............................................................
PI96354 ............................................................

Williams82 ACGCACGCAAGCCAAACGAAAATCAGAAGAACTTGTTCATGGCACAAGGCAGAGAGGACC
Forrest ............................................................
PI96354 ............................................................

Williams82 CTAACCAAAACACTGGCATATCCATCTTGAACTGCAAGATTGCAGCTGCTGCAGATTTGA
Forrest ............................................................
PI96354 ............................................................

Williams82 TCCCTGTGAAATCCTCGTTCAAGAGCTACCTAGGACGTCCTTGGAAAATGTACTCTATGA
Forrest ............................................................
PI96354 ............................................................

Williams82 CTGTTGTGTTAAAATCTTACGTGGATATAGACCCAGCAGGGTGGTTGGAATGGAATGAAA
Forrest ............................................................
PI96354 ............................................................

Williams82 CATTTGCATTGGATACGTTGTATTATGGGGAGTACATGAATAGGGGTCCATGTTCAAACA
Forrest ............................................................
PI96354 ............................................................

Williams82 CAAGTGGTAGGGTTACGTGGCCAGGTTATAGGGTCATTAACAGCTCCATTGAGGCAAGCC
Forrest ............................................................
PI96354 ............................................................

Williams82 AATTCACAGTTGGACAGTTTATTCAAGACAATGATTGGTTGAACAACACTGGCATCCCAT
Forrest ............................................................
PI96354 ............................................................

Williams82 TCTTCTCTGGTTTGAGTTGAGAATATTTTTAGGTTGAGCTGAACCTAGGAGATCACGTGA
Forrest ............................................................
PI96354 ............................................................

Williams82 TTTGTAAGGTTACATTACATTACATTCCTGTGTATTACTTCATTGGAGTTCCTATAGTAT
Forrest ............................................................
PI96354 ............................................................

Williams82 AGGGCTATTGTGGCTATAGCAGTCACGTGATGACTATACCCAAAAATTAGTCTTGTGAGG
Forrest ............................................................
PI96354 ............................................................

Williams82 GATTATATATTTTAGTGCTCTAATAAAAGCAACTAACCCCACCAGAGATAT
Forrest ...................................................
PI96354 ...................................................

## Glyma.10G017100 Protein Alignment

Since Forrest and PI 96354 sequence are identical, only Forrest is shown in the following alignment. Likewise, as Essex, Bossier, and Williams82 sequences are identical, only Williams82 is shown in the following alignment.

Williams82 MNNLTLASILTVISSLLFFGTTHLTNTQTTRVPDQKHKHLHFQKHIQVVAQSTCEGTLYP
Forrest ............................................................

Williams82 DLCVLTLATFPDLTTKSVPQVISSVVNHTMYEVRSTSYNCSGLKKMLKNLNPLDQRALDD
Forrest ................................I..........................Y

Williams82 CLKLFEDTSVELKATIDDLSIKSTIGSKLHHDLQTLLSGAMTNLYTCLDGFAYSKGRVGD
Forrest ............................R.................Y.............

Williams82 RIEKKLLQISHHVSNSLAMLNKVPGVEKLTTSSESDEVFPEYGKMQKGFPSWVSSKDRKL
Forrest .................................D..........................

Williams82 LQAKVNETKFNLVVAKDGTGNFTTIGEALSVAPNSSTTRFVIHVTAGAYFENVEVIRKKT
Forrest .*

Williams82 NLMFVGDGIGKTVVKGSRNVEDGWTIFQSATVAVVGAGFIAKGITFEKSAGPDKHQAVAL
Forrest

Williams82 RSGVLTSQLSTNAVSLATRTLSTSIPCANSTVNVTFMAL*
Forrest
